# Evolution of Southern Hemisphere Westerly asymmetry since the Early Miocene

**DOI:** 10.1126/sciadv.aee0530

**Published:** 2026-05-27

**Authors:** Congcong Gai, Torben Struve, Andrew P. Roberts, Yiwen Li, Chenguang Zhu, Yanhong Chen, Jilin Wei, Wei Liu, Weijie Zhang, Hailong Liu, Huaichun Wu, Qingsong Liu

**Affiliations:** ^1^ State Key Laboratory of Geomicrobiology and Environmental Changes, China University of Geosciences, Beijing 100083, China.; ^2^ Key Laboratory of Polar Geology and Marine Mineral Resources [China University of Geosciences (Beijing)], Ministry of Education, Beijing 100083, China.; ^3^ Marine Isotope Geochemistry, Institute for Chemistry and Biology of the Marine Environment (ICBM), University of Oldenburg, Oldenburg 26129, Germany.; ^4^ Research School of Earth Sciences, Australian National University, Canberra ACT 2601, Australia.; ^5^ Institute of Atmospheric Physics, Chinese Academy of Sciences, Beijing 100029, China.; ^6^ Centre for Marine Magnetism (CM^2^), Department of Ocean Science and Engineering, Southern University of Science and Technology, Shenzhen 518055, China.; ^7^ Frontiers Science Center for Deep-time Digital Earth, China University of Geosciences (Beijing), Beijing 100083, China.

## Abstract

The Southern Hemisphere Westerlies (SHW) are a crucial component of Earth’s climate system because they regulate moisture, temperature, and ocean circulation throughout the Southern Hemisphere. They also affect the Northern Hemisphere through atmospheric and oceanic teleconnections. Yet, their variations are poorly constrained for past warm climate intervals that could serve as analogs for possible future climate evolution. Here we present geochemical and magnetic data from South Pacific Ocean sediments to reconstruct dust provenance since the Early Miocene. We find that the eolian dust source shifted from Central South America to Australia at ~8.4 million years ago. Our climate simulations also suggest that a strong middle Miocene subtropical jet facilitated dust transport from Central South America to the South Pacific Ocean. If the middle Miocene is used as a possible analog for future warming, we suggest that the SHW tends to be more zonally asymmetric in warm climate states and that SHW asymmetry may be important for future climate projections.

## Teaser

A westerly split jet dominating Miocene South Pacific dust deposition was superseded by a single jet configuration 8.4 Ma ago.

## INTRODUCTION

The Southern Hemisphere Westerlies (SHW) play a central role in controlling Earth’s large-scale extratropical moisture and temperature distribution (*1*, *2*). They affect atmospheric carbon dioxide contents by modifying both the upwelling and subduction/convection branches of deep and intermediate waters in the Southern Ocean (*3*, *4*), as well as by modulating productivity via addition of dust-derived micronutrients (*5*, *6*), which makes them an essential variable in climate projections (*7*, *8*). The SHW are usually regarded as zonally symmetric (*9*). However, meteorological reanalysis indicates a statistically insignificant northward SHW shift over the South Pacific Ocean and a significant southward shift over the Atlantic and Indian Oceans over recent decades (*10*), implying zonally asymmetric SHW behavior. Moreover, the SHW differ across ocean basins in austral winters, with the upper-level SHW core splitting into subtropical and subpolar branches over the South Pacific Ocean while behaving as a single mid-latitude jet across the Atlantic and Indian Oceans (*11*). Considering this complexity, reconstructing SHW variability in time and space, especially over the South Pacific Ocean, is crucial for understanding their underlying dynamics and predicting future climate change.

**Fig. 1.**
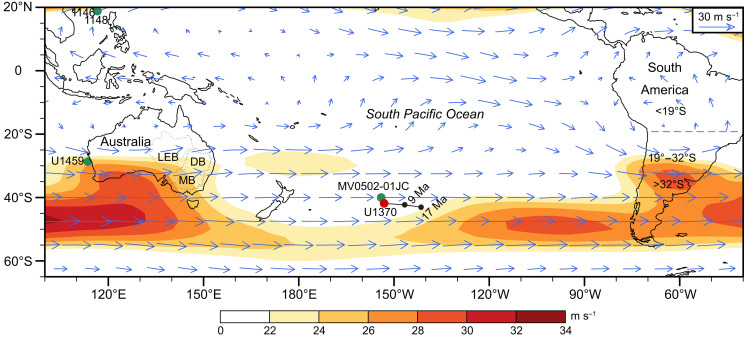
Schematic map of the South Pacific Ocean and surrounding regions. The present-day locations of Site U1370 (41°51.12′S, 153°6.38′W, red dot) and other cores mentioned in this study are indicated. The backtrack path of Site U1370 was reconstructed using the Gplates open source software and plate reconstruction in (*77*); black dots along the backtrack path denote locations of Site U1370 at 17 and 9 Ma. Blue arrows and shading indicate the austral summer wind vector (unit: meters per second) and locations with maximum winds at 200 hPa between 1979 and 2023 from the NCEP/DOE Reanalysis II data (https://psl.noaa.gov), respectively. MB, Murray Basin; DB, Darling Basin; LEB, Lake Eyre Basin.

Proxy-based SHW reconstructions over the South Pacific have focused on the Late Quaternary and indicate that the SHW shifted southward and/or strengthened during cold intervals on millennial timescales (*12*), while the upper-level SHW split into subtropical and subpolar jets over the South Pacific and the subtropical jet intensified during precession maxima on orbital timescales (*13*). However, the Late Quaternary was colder than present. To better predict future climate scenarios under global warming conditions, it is crucial to investigate the response of the SHW in warmer-than-present climate states. The Miocene [~23–5.3 million years (Ma) ago] is a promising analog, where extreme climate changes occurred under global warmth such as the Miocene Climatic Optimum (~16.9 to 14.7 Ma), which was 7° to 8°C warmer than today (*14*), while other boundary conditions are close to those of today (*15*). Apart from the lack of studies during warm geological periods, available reconstructions mainly use precipitation and moisture proxies to infer wind changes (*12*, *13*, *16*), while there is a paucity of proxies related directly to wind transport. Furthermore, the relationship between paleo-precipitation and SHW is not always straightforward, which can lead to contradictory paleoclimate interpretations (*17*, *18*). Here, we use rock magnetic and geochemical proxies, specifically the strontium (Sr) and neodymium (Nd) isotope signatures of wind-related dust proxies, to assess of eolian dust characteristics at mid-latitude Central South Pacific Ocean Site U1370 (Fig. 1), combined with climate simulations to explore Miocene and Pliocene SHW dynamics.

## RESULTS AND DISCUSSION

### Dust provenance changes in the Central South Pacific

To constrain the origin of mineral dust particles at Site U1370, the <5-μm fraction was chosen for Sr and Nd isotopic analyses. Site U1370 bulk sediment contains components of different origin (*19*). Our physical and chemical pretreatment extracted the lithogenic <5-μm fraction (Materials and Methods, text S1, and figs. S1 and S2). The <5-μm fraction was chosen because it is mainly of eolian origin (text S1), its long atmospheric lifetime allows tracing of dust particles over long distances, and its high cohesiveness makes this size fraction less susceptible to postdepositional reworking by ocean currents (*20*, *21*).

**Fig. 2.**
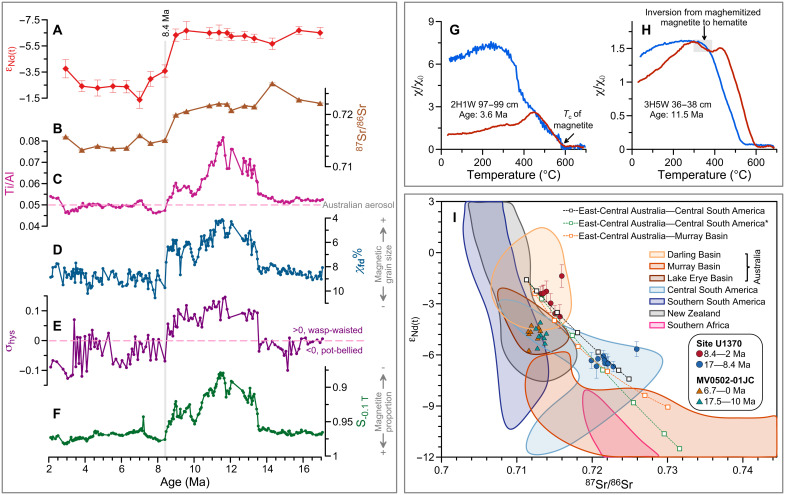
Rock magnetic and geochemical results from Site U1370. (**A**) ε_Nd_ variations (2σ error bars). (**B**) ^87^Sr/^86^Sr variations (error bars smaller than symbols). (**C**) Ti/Al. The dashed line denotes the value for modern Australian aerosol samples (0.050) in (*30*). (**D**) χ*_fd_*%. (**E**) Hysteresis loop shape parameter, σ_hys_. The dashed line denotes the division between wasp-waisted and pot-bellied hysteresis shapes. (**F**) S_-0.1 T_. The gray line in (A) to (F) denotes 8.4 Ma. (**G**) χ-T curve for a sample deposited at 3.6 Ma. The black arrow denotes the Curie temperature of magnetite (*T*_c_, ~585°C). (**H**) χ-T curve for a sample deposited at 11.5 Ma. Magnetic susceptibility loss during heating between ~300° and 400°C (shaded area) is interpreted as the inversion from maghemitized magnetite to hematite. Red (blue) lines in (G) and (H) are heating (cooling) curves. (**I**) Comparison of Site U1370 fine-grained (<5 μm) sediments with potential Southern Hemisphere source areas and Marlin Rise core MV0502-01JC sediments (*44*). Note that Australian and Southern African data extend beyond the scale of the figure. Mixing between important source regions is indicated by dashed lines with white squares; squares from left to right indicate 100, 90, 70, 50, 30, 10, and 0% of the East-Central Australian source. Central South American* reflects the “extreme” Central South American end-member composition as described in the main text. See tables S1 and S2 for source area and end-member data. Error bars denote the 2σ SD.

The Site U1370 Sr-Nd isotope data are distributed linearly (Fig. 2I), as expected for binary mixing of end-members with different Sr-Nd isotope characteristics. On the basis of geological and climatic conditions, we exclude Antarctica, southern Africa, and New Zealand as major suppliers of the <5-μm fraction material over the investigated 17- to 2-Ma interval (text S2). Australia is a major dust source in the modern Southern Hemisphere and is geochemically diverse (*22*). On subcontinental scale, the main potential source areas are the Murray-Darling Basin and the Lake Eyre Basin (*22*). On the basis of geochemistry and hydrology, the Murray-Darling Basin in southeastern Australia can be separated into the Murray and Darling basins (Fig. 1). Site U1370 radiogenic isotope data for samples deposited before 8.4 Ma are similar to the Murray Basin (ε_Nd_ = −9.1 ± 4.0, ^87^Sr/^86^Sr = 0.7300 ± 0.0334, 2SD, *n* = 24) (*23*–*25*), while samples deposited since 8.4 Ma form a narrow cluster in Sr-Nd isotope space with radiogenic isotope compositions (ε_Nd_ = −2.8 ± 1.1, ^87^Sr/^86^Sr = 0.7142 ± 0.0018, 2SD, *n* = 7) more similar to compositions found in the Darling (ε_Nd_ = −1.6 ± 3.0, ^87^Sr/^86^Sr = 0.7113 ± 0.0032, 2SD, *n* = 11) and Lake Eyre basins (ε_Nd_ = −3.9 ± 1.8, ^87^Sr/^86^Sr = 0.7110 ± 0.0030, 2SD, *n* = 24) (Fig. 2I and table S1) (*23*–*26*). Therefore, a hypothetical mixing scenario between the Murray Basin and the Darling and/or Lake Eyre basins may explain the Sr-Nd characteristics at Site U1370 (Fig. 2I). Magnetic minerals in Site U1370 samples deposited before 8.4 Ma are dominated by low-coercivity magnetite and medium-coercivity maghemitized magnetite (Fig. 2, G and H), while the Murray and Darling basins do not host the latter (*27*, *28*), which excludes mixing between these two source regions. Although co-occurring magnetite and maghemitized magnetite have been observed in the Lake Eyre Basin (*29*), lower ε_Nd_ and higher ^87^Sr/^86^Sr corresponding to higher HIRM_-0.1 T_ and lower S_-0.1 T_ before 8.4 Ma (and vice versa after 8.4 Ma) also exclude mixing between the Murray and Lake Eyre basins to explain the Late Miocene dust provenance shift at Site U1370 (Fig. 2, A, B, F, and I; fig. S3B; and text S3). Therefore, dust mixing from different Australian basins alone cannot explain our data. However, the Darling and Lake Eyre basins are identified as a high-ε_Nd_, low ^87^Sr/^86^Sr end-member in a binary mixing scenario. The Nd-Sr isotope data suggest that this end-member is dominated by the Darling Basin, consistent with low HIRM_-0.1 T_ and higher S_-0.1 T_ indicating a higher proportion of magnetite in the post–8.4 Ma samples (Fig. 2, F, G, and I, and figs. S3B and S4A). Moreover, taking East-Central Australia (i.e., the Darling and Lake Eyre basins) as a high-ε_Nd_, low ^87^Sr/^86^Sr end-member also explains modest titanium/aluminum (Ti/Al) variability around ~0.049 g/g since 8.4 Ma, which is similar to that for modern Australian aerosol samples (~0.050 g/g) (Fig. 2C, fig. S5, and text S4) (*30*).

In South America, potential dust source areas are located in a long and narrow region extending from the coast of Peru to the coast of Patagonia. Patagonia (>32°S) is climatically dominated by the SHW and has been identified as a major Southern Hemisphere dust source (*31*). Pleistocene dust emission variations from Patagonia have been linked with glacial activity (*32*). Subhumid to humid conditions in the Early to middle Miocene were followed by the onset of glaciation in Patagonia after ~10 Ma (*33*, *34*). However, even under peak glacial conditions, only a very small fraction of Patagonian dust reached the South Pacific (*35*, *36*), which excludes it as a potential end-member (table S1). In contrast, Sr-Nd data for Site U1370 samples deposited before 8.4 Ma mainly fall within the range of sediment compositions in Central South America (~19° to 32°S) (Fig. 2I) (*37*). After excluding other potential source regions as a low-ε_Nd_, high ^87^Sr/^86^Sr end-member, mixing of dust from East-Central Australia and Central South America can explain our data (Fig. 2I). It has been shown previously for the Late Pleistocene that dust from Central Australia and Central South America reached the South Pacific Ocean (*35*, *36*). Furthermore, our mixing scenario is consistent with the magnetic mineral properties of Site U1370 sediments showing the co-occurrence of (titano)magnetite and maghemitized (titano)magnetite, which are the dominant magnetic minerals in Central South American loess-paleosol and sediment sequences (Fig. 2, E and H, and fig. S3) (*38*, *39*). The shift in dust provenance at ~8.4 Ma is ascribed to an abrupt reduction of Central South American dust (Fig. 2I), as also reflected by the shift in the grain size of magnetic particles and maghemitized magnetite concentrations (Fig. 2, D to F, and fig. S3). The abrupt nature of the shift excludes plate tectonic movements as a primary control on the dust provenance signal at Site U1370 (text S5).

Ti/Al changes do not exactly correspond with the Nd isotope record, likely documenting both the dust provenance shift and changing source region weathering conditions. More specifically, we observe a shift in Ti/Al and magnetic parameters between ~14 and 8.4 Ma (Fig. 2, C to F), which we ascribe primarily to changes in Central South American weathering conditions. Weathering in Central South America peaked between 25 and 14 Ma and then declined (*40*, *41*), leading to different Ti/Al ratios before and after ~14 Ma. Before ~14 Ma, Al was leached more readily from parent material than Ti during intense chemical weathering (*42*), implying an enrichment of Al in the sedimentary products (i.e., dust). This would explain the lower Ti/Al values in the oldest part of our record, their subsequent increase and the overall rather invariably Nd isotope composition in the dust fraction of Site U1370. Reduced chemical weathering in Central South America after 14 Ma would also impede pedogenic fine-grained magnetic mineral formation, consistent with a predominant deposition of coarse magnetic minerals as indicated by the relatively low χ*_fd_*% and anhysteretic remanent magnetization (ARM)/saturation isothermal remanent magnetization (SIRM) values between ~14 and 8.4 Ma at Site U1370 (Fig. 2D and fig. S3A). These changes in chemical weathering conditions may have been related to the uplift of the Puna Plateau in the central Andes at ~15 ± 1.2 Ma, which blocked easterly moisture supply (*43*). Our data extend the existing Late Pleistocene provenance records to the middle Miocene and indicate a pronounced shift in chemical weathering intensity in the Central South American source regions during the middle to Late Miocene.

The calculated mixing trend between Australian and Central South American source areas reveals that Central South America contributed more than 70% to our study area in the Early to Late Miocene with a primary dust source shift to Australia at 8.4 Ma (Fig. 2I). We acknowledge that many of the isotope data for the Central South American source region show a wide compositional range (*37*), and the average may be not representative of the variation. Therefore, we tested a hypothetical “extreme” Central South American end-member scenario in which the Central South American dust contribution decreased from ~50 to ~20% across the 8.4-Ma transition (Fig. 2I), which indicates that the observed dust source shift does not entirely depend on the choice of end-member composition (text S2). We further compare our results with Sr-Nd isotope data from nearby core MV0502-01JC (*44*). The core MV0502-01JC data are close to our calculated mixing line, with overall less radiogenic Sr isotopic compositions (Fig. 2I). This can be attributed to differences in grain size composition affecting primarily the Sr isotope composition, possible incomplete removal of authigenic Nd and Sr carrying phases during leaching, and/or age model imprecision (text S6).

**Fig. 3.**
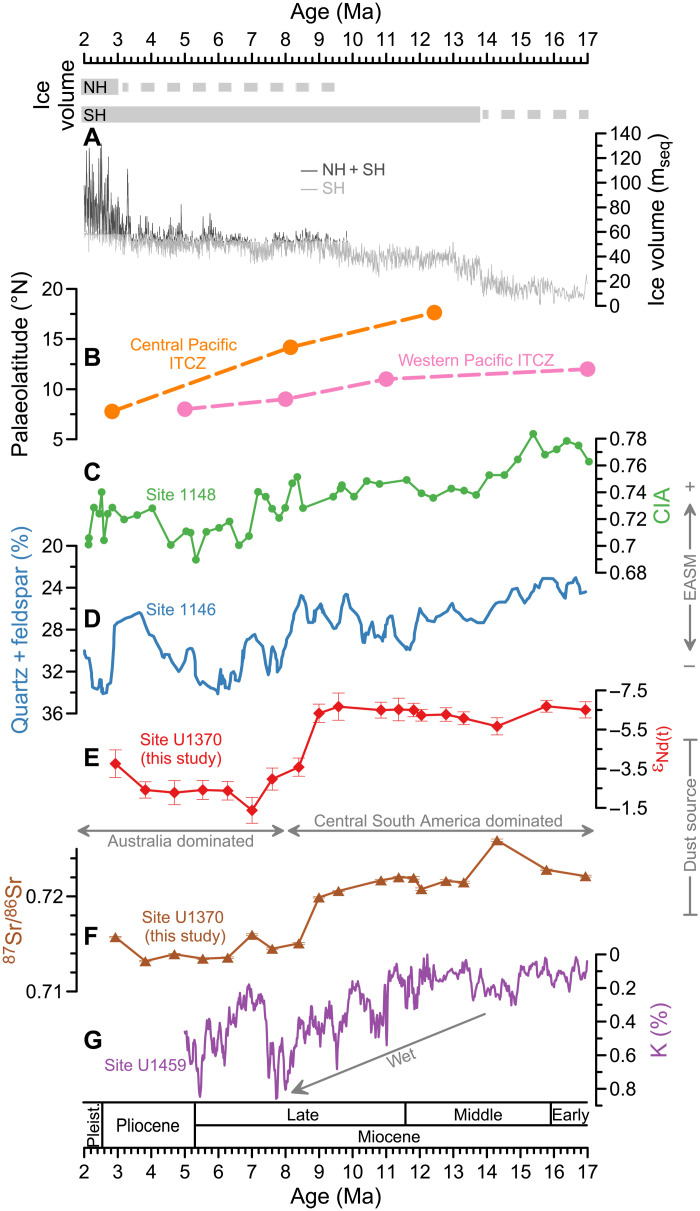
Comparisons of global climate changes with Sr-Nd isotopic data from Site U1370 over the 17- to2-Ma time interval. (**A**) Estimated ice volume in m_seq_ for the SH (light gray) and NH (dark gray) (*56*). Horizontal bars denote ice volume, with the dashed bar representing periods of small-to-mid size ice volume, and the full bar representing close to full size ice volume. (**B**) Reconstructed marine ITCZ trajectories. Orange dots are from Ocean Drilling Program (ODP) Leg 199, central Pacific Ocean (*53*); pink dots are from western Pacific Ocean (*54*). (**C**) Chemical index of alteration (CIA) values from ODP Site 1148, South China Sea (*52*). (**D**) Weight percent of (quartz + feldspar) in terrigenous minerals from ODP Site 1146, South China Sea (*51*). (**E**) ε_Nd_ variations (2σ error bars) at Site U1370 (this study). (**F**) ^87^Sr/^86^Sr variations (error bars smaller than symbols) at Site U1370 (this study). (**G**) Wireline log K data from IODP Site U1459, southwest Australia (*16*). m_seq_, meter sea level equivalent. The dust source is classified as “Australia dominated” (black dashed line in Fig. 2I) when the East-Central Australian end-member contributes more than 50% (ε_Nd_ > −4.7 and ^87^Sr/^86^Sr < 0.7181), and “Central South America dominated” when the Central South American end-member contributes more than 50% (ε_Nd_ < −4.7 and ^87^Sr/^86^Sr > 0.7181). NH, Northern Hemisphere; SH, Southern Hemisphere; EASM, East Asian summer monsoon.

Mixing results could also be affected by dilution effects, i.e., more supply from one source can be caused by reduction from another. Dilution effects cannot be estimated accurately because of the lack of quantitative dust flux constraints (*36*). Here, we qualitatively assess source area conditions because they are associated with dust generation, which may affect dust flux and reconstructed relative source area contributions. Arid conditions have prevailed at least since the middle Miocene in Central South America (*45*) and have probably been promoted by the uplift of the Puna Plateau reducing moisture supply and increasing physical weathering from ~15 ± 1.2 Ma (*43*). These conditions increased sediment availability for deflation. At the same time, Australia experienced relatively humid conditions, as indicated by intensified precipitation and fluvial runoff (Fig. 3G) (*16*). Although humid environments favor sediment recharge in Australian dust sources, dust entrainment and transport processes were subdued (*46*). Therefore, the high Central South American proportion was possibly related to low Australian contributions before 8.4 Ma ago (Fig. 2I). More arid conditions in both Central South America and Australia then led to a more balanced dust provenance signal in the South Pacific after 8.4 Ma ago (*16*, *33*).

### Dust transport dynamics since the Early Miocene

Dust provenance shifts are often associated with atmospheric circulation variations (*35*). Atmospheric circulation over Central South America is dominated by the trade winds and SHW. The southeasterly trade winds prevail in the lower troposphere and transport dust to lower-latitude regions such as the Equatorial Pacific Ocean rather than to our study area (*47*). Instead, the westerly jet, which prevails in the middle and upper troposphere, is the primary transporter of long-distance Central South American dust transport along a circumpolar route to the South Pacific Ocean (*35*).

**Fig. 4.**
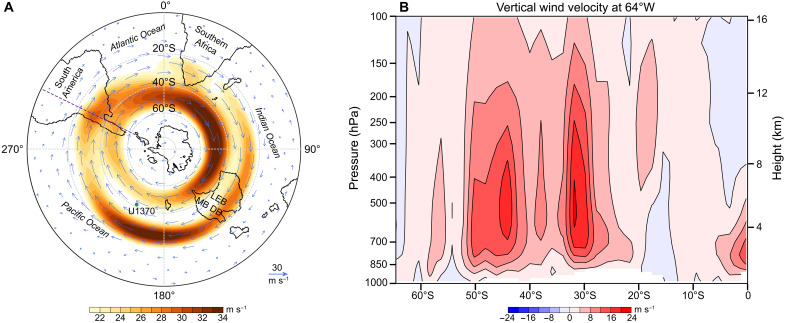
Simulated Miocene 100-year annual mean of Southern Hemisphere atmospheric circulation (with 560 ppm *p*CO_2_). (**A**) Wind vector at 200 hPa. The SHW core splits into subtropical and subpolar jets over the South Pacific Ocean. Shading indicates locations with maximum wind speeds. Blue arrows indicate the wind vector (unit: meters per second); the green dot indicates the present-day location of Site U1370; the purple dashed line indicates the location of the cross section shown in (B). (**B**) Latitude-pressure cross section of vertical wind velocity (filled colors, unit: meters per second) at 64°W. Positive (negative) values represent upward (downward) velocity.

**Fig. 5.**
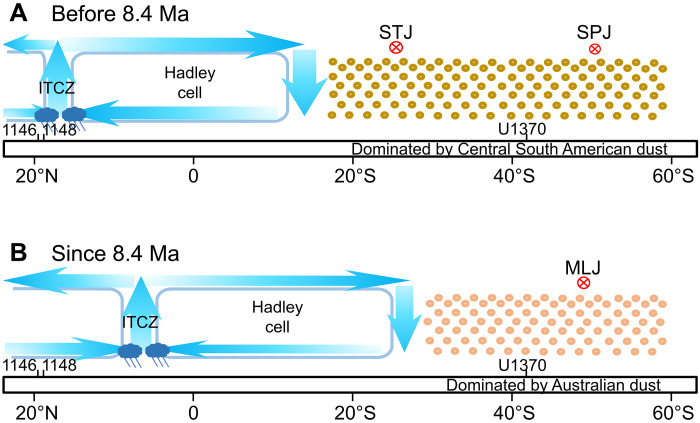
Schematic meridional profiles of relative atmospheric circulation pattern changes. Present-day locations of Sites 1146, 1148, and U1370 are indicated at the respective latitudes. (**A**) Before 8.4 Ma, the ITCZ was situated in a relatively northward position, with the SHW core over the South Pacific split into a strong subtropical jet, which favors Central South American dust transport to the South Pacific Ocean, and a subpolar jet. (**B**) Since 8.4 Ma, the ITCZ moved southward; the SHW over the South Pacific is characterized by a mid-latitude jet that transports more Australian dust to the South Pacific Ocean. Circled crosses indicate westerly winds transporting into the page. STJ, subtropical jet; SPJ, subpolar jet; MLJ, mid-latitude jet.

To explain mechanistically the dust provenance shift over the Central South Pacific at ~8.4 Ma ago, we evaluated climate simulations forced by 560 and 280 parts per million (ppm) CO_2_, respectively. Dust from both Australian and Central South American source regions is deposited at the same time, that is, within the temporal resolution of our individual sample (2 cm), and Site U1370 sedimentation rate of 120 to 280 cm/Ma is overall low, excluding that a seasonal signal is resolved at Site U1370. Therefore, climate states analyzed here are based on the average over the final 100 model simulation years (see Materials and Methods). As shown in Fig. 4A and fig. S6A, the SHW at high altitude in both simulations splits into strong subtropical and subpolar jets from the South Pacific to the east of South America. The simulated 100-year annual mean westerly jet structure over the South Pacific is similar to that in the modern austral winter and differs from that in other seasons (*11*, *48*), which further indicates a different dust transport pathway compared to the present day and the Holocene. Also, the vertical wind direction over the Central South American source area is upward throughout the troposphere (Fig. 4B and fig. S6B), which implies that Central South American dust can be lofted to subtropical jet heights for long-distance transport. The strength and position of the subtropical jet is associated with the Hadley cells, which can be altered substantially by meridional intertropical convergence zone (ITCZ) shifts (*49*). Low-latitude monsoon climate is tightly coupled with ITCZ migration (*50*). A stronger East Asian summer monsoon before ~8 Ma indicates that the Miocene ITCZ was situated in a more northerly position (Fig. 3, C and D) (*51*, *52*), consistent with ITCZ migrations identified both in western and central Pacific regions (Fig. 3B) (*53*, *54*). Moreover, climate simulations suggest that intensified high southern latitude ice cover cools and dries the entire southern high- and mid-latitudes through atmospheric advection, which leads to a northward progression of cooler sea surface temperature anomalies and further shifts marine ITCZ northward (*55*). Antarctic ice sheets were dynamic in the Early Miocene with major Antarctic glaciation development from ~14 to 8 Ma (*56*). This kept the Early-to-Late Miocene ITCZ in a more northerly position, thus weakening the northern and strengthening the southern Hadley cell (*48*). As a result, the South Pacific subtropical jet intensified and shifted to lower latitudes (Figs. 4A and 5A and fig. S7), thus transporting more dust from Central South America to the South Pacific, while dust emissions from Patagonia were unlikely to have reached the South Pacific (see also above in the “Dust provenance changes in the Central South Pacific” subsection) (*34*).

With Late Miocene cooling, Northern Hemisphere continental ice sheets began to develop (Fig. 3A) (*56*, *57*), which cooled the Northern Hemisphere, leading to equatorward ITCZ migration and a weakened (intensified) East Asian summer (winter) monsoon (Fig. 3, B to D) (*51*, *52*). Weakening of the southern Hadley cell would have weakened the South Pacific subtropical jet (*48*). The mid-latitude jet became stronger again (Fig. 5B) and would have transported predominantly Australian dust to Site U1370, as indicated by Sr-Nd isotopes (Fig. 2I).

Our magnetic and geochemical data suggest that dust in the Central South Pacific was predominantly from Central South America rather than Australia from 17 to 8.4 Ma. Climate simulations and complementary geological evidence further reveal that dry conditions in the Central South American dust regions and long-range dust transport from this area are favored by a strong subtropical jet over the South Pacific, which is associated with a more northerly Early-to-Late Miocene ITCZ position. Over the past two decades, the ITCZ latitude has shifted northward with global warming at ~0.3°/decade for land and ~ 0.15°/decade for ocean (*58*). If this rate continues, the zonal SHW asymmetry will increase when taking the middle Miocene as predictive of future warming, i.e., to produce strong subtropical and subpolar jets over the South Pacific Ocean. Wind stress pattern changes have substantial implications for global ocean circulation. At mid-latitudes, the Southern Ocean is an important CO_2_ sink, absorbing ~40% of anthropogenic CO_2_ emissions (*59*). Climate simulations suggest that wholesale SHW intensification and/or poleward shifts weaken the Southern Ocean CO_2_ sink by enhancing deep ocean ventilation and increasing circumpolar deep water upwelling (*3*, *60*). However, these studies often underestimate or neglect asymmetric SHW behavior over the South Pacific Ocean. As a result, it is unknown how the SHW split modified surface and deep circulation, and how this affected Southern Ocean CO_2_ accumulation. Moreover, asymmetric SHW switch the dust source in the South Pacific Ocean, as indicated by our results. Dust-borne Fe is important for supporting phytoplankton productivity in the Fe-limited mid-latitude South Pacific (*5*, *61*). Previous studies suggest that Central South American dust-Fe is chemically more pristine and possibly more bioavailable than Australian dust (*35*, *36*, *62*, *63*). If the dilution effects were negligible, reduced input from Central South America would have reduced the supply of bioavailable Fe. Despite different topography, vegetation, ice-sheet extent, and ocean gateway configuration, the middle Miocene is considered an analog for future high-CO_2_ warming (*15*, *64*). Our findings provide insights into linked changes of SHW asymmetry and climate evolution during this important interval. Furthermore, it remains to be explored whether the zonal SHW asymmetry is limited to the South Pacific Ocean or whether it also exists in the Indian sector of the Southern Ocean as indicated by our model results. Therefore, we suggest that SHW asymmetry should be incorporated in future climate projections of global warming.

## MATERIALS AND METHODS

### Materials and age model

The study area is in the South Pacific Ocean where sediment cores were collected during the Integrated Ocean Drilling Program (IODP) Expedition 329. Site U1370 is located at 41°51.12'S, 153°6.38'W (5074.2-m water depth, Fig. 1), and is surrounded by abyssal hills and seamounts. A total of 223 samples (between 4.08 and 32.96 m below seafloor, mbsf) were taken from Hole 1370E. Sediments are dominated by red-brown to yellow-brown zeolitic metalliferous pelagic clay (*65*). A Co-based age model with nondetrital Co flux of 4176 μg/cm^2^ per million years (Myr) (*66*) was used for Site U1370. Sample ages are calculated by A_i_ = A_c_ + (D_i_ - D_c_)/S_i_, where A_i_ and A_c_ are ages of the sample and the age constraint, respectively, and D_i_ and D_c_ are depths of the sample and age constraint, respectively, and S_i_ is the sedimentation rate. Detailed information on age constraints and sedimentation rates are provided in (*66*). A baseline uncertainty of the Co method is considered when calculating the age uncertainty of samples.

### Rock magnetic analyses

Magnetic remanence measurements for all samples were made using a 2-G Enterprises 760R cryogenic magnetometer with in-line alternating field (AF) demagnetizer. An ARM was imparted in a 100-mT peak AF with a 0.05-mT direct current bias field. Isothermal remanent magnetizations (IRMs) were imparted at 1 and −0.1 T, which are defined as the SIRM and *IRM_-0.1 T_*, respectively. The “hard” IRM (HIRM) is defined by HIRM_-0.1 T_ = (SIRM + *IRM_-0.1 T_*)/2 (*67*). S_-0.1 T_ is defined as S_-0.1 T_ = (1 − *IRM_-0.1 T_*/SIRM)/2 (*68*). The HIRM_-0.1 T_ and SIRM flux were calculated by multiplying by the mass accumulation rate (MAR), which is defined as MAR = LSR × DBD, where LSR is the linear sedimentation rate (in centimeters per million years) and DBD is the dry bulk density (in grams per cubic centimeter).

Low-field magnetic susceptibility (χ, mass specific) was measured for all samples at frequencies of 976 and 15616 Hz. The percentage absolute frequency-dependent susceptibility is calculated by χ*_fd_*% = (χ*_976 Hz_* − χ*_15616 Hz_*)/χ*_976 Hz_* × 100%. Temperature-dependent magnetic susceptibility (χ-T) curves were measured at a 976 Hz from room temperature to 700°C in an argon atmosphere for representative samples. χ and χ-T curves were measured using an AGICO Kappabridge MFK 2-FA system equipped with a CS-3 furnace.

Hysteresis loops were measured for every second sample using a Lakeshore 8600 vibrating sample magnetometer. Saturation magnetization (*M*_s_) and coercivity (*B*_c_) were obtained from hysteresis loops. The shape parameter (σ_hys_) is defined by $\sigma_{\text{hys}}=\text{log}\left\{\int_{-B_{\text{max}}}^{B_{\text{max}}}\left[M^{+}\left(B\right)-M^{-}\left(B\right)\right]\mathrm{dB}/4M_{s}B_{c}\right\}$ (*69*), where *B*, *M*^+^(*B*), and *M*^−^(*B*) represent the applied field and upper and lower hysteresis loop branches, respectively. Values of σ_hys_ for pot-bellied (wasp-waisted) loops are below (above) zero (*69*).

### Grain-size analyses

Before undertaking grain-size analyses, samples were treated successively with 5% H_2_O_2_ and 0.5 M HCl in a water bath at 85°C to remove organic matter, carbonates, and Fe-Mn oxides, respectively. No steps aiming to remove silica microfossils were taken because the studied sediments are dominated by red clay and lack biogenic opal (*70*). The sediment solution was then rinsed and centrifuged to a near-neutral pH condition, deflocculated in 0.05 M sodium hexametaphosphate, ultrasonicated for 5 min, and followed by measurement using a Malvern Mastersizer 3000 Laser Particle Size Analyzer. Each sample was measured three times in the 0.01- to 3500-μm size range; the average spectrum for the three measurements was used to represent the grain size distribution. End-members were fitted with a General Weibull distribution using the AnalySize software package (*71*), and 1 to 10 end-members were fitted to the whole dataset.

### Geochemical analyses

Contents of major elements (Ti and Al) were measured using a Varian 720 inductively coupled plasma (ICP) optical emission spectrometer. Before measurement, samples were digested with HF-HNO_3_ (3:1) in closed Teflon bottles and heated at 180°C for 12 hours, then dried, and finally redissolved with 6 M HCl and heated at 150°C for 12 hours. Standard (GBW07314, GBW07315, GBW07316, BHVO-2, and BCR-2), blank, and replicate measurements were performed to assess analytical precision and accuracy. Standard reference materials were measured three times. The average 2 relative standard deviation (RSD) precision of the repeat analysis for standard reference material was ~2.0% for Ti and 0.9% for Al in this study. The accuracy of standard reference measurements was better than 7.0% for Ti and 1.4% for Al compared to reported reference values.

Bulk sediments were wet-sieved with subsequent Stokes-based separation to extract the <5-μm grain-size fraction. The <5-μm fraction was then treated with 5% H_2_O_2_ to remove organic matter followed by 1 M HCl to remove carbonate and Fe-Mn oxides. After that, samples were triple-rinsed with Milli-Q water and freeze-dried. Freeze-dried samples (~80 to 100 mg) were treated with HF-HNO_3_ (1:1) at 120°C. After complete digestion, the samples were dried and redissolved in HNO_3_ and were finally completely dried. The radiogenic Sr and Nd isotope compositions were determined using a Thermo Fisher Scientific Neptune Plus multicollector ICP–mass spectrometry. For Sr isotope analyses, mass bias was corrected using ^86^Sr/^88^Sr = 0.1194. Replicate analyses of the NIST SRM 987 reference material were performed every five samples to correct for instrumental offset from the reported NIST SRM ^87^Sr/^86^Sr value of 0.710248 (*72*). The external reproducibility of W-2a and BHVO-2 was ^87^Sr/^86^Sr = 0.70696 ± 0.00002 and 0.70345 ± 0.00002 (2SD, *n* = 9), respectively, which are consistent with reported values in (*73*). For Nd isotope analyses, mass bias was corrected using ^146^Nd/^144^Nd = 0.7219. Replicate analyses of the JNdi-1 reference material were performed every five samples to correct for instrumental offset from the reported JNdi-1^143^Nd/^144^Nd value of 0.512115 ± 0.000007 (*74*). The external reproducibility of W-2a and BHVO-2 was ^143^Nd/^144^Nd = 0.512683 ± 0.000011 (2SD, *n* = 4) and 0.512641 ± 0.000009 (2SD, *n* = 4), respectively, which are consistent with reported values in (*73*). Results are expressed in epsilon notation ε_Nd_ = [(^143^Nd/^144^Nd)_sample_/(^143^Nd/^144^Nd)_CHUR_ − 1] * 10^4^, where CHUR is the Chondritic Uniform Reservoir (*75*). The total chemical procedure blanks for Sr and Nd were below 0.65 and 0.08 ng, respectively. Blank contaminations were significantly <1% of the individual sample Sr and Nd and have negligible effects on the respective sample isotope compositions.

### Climate simulations

Our numerical simulations use a fully coupled general circulation model FGOALS-g3, which is extensively described in (*76*). It comprises the atmospheric model GAMIL3, ocean model LICOM3, land model CAS-LSM, sea-ice model CICE4, and river transport model (RTM). GAMIL3 and CAS-LSM share rectilinear coordinates with 2° horizontal resolution, while LICOM3 and CICE4 share a tripole grid with 360 × 218 grid cells. For the vertical coordinate, a sigma coordinate with 26 layers is used in GAMIL3, and the η vertical coordinate with 30 layers is used in LICOM3. In RTM, water is conveyed from each land grid cell in the downstream direction and ultimately to the neighboring ocean grid cell. Communications among these components are achieved via the CPL7 flux coupler from the National Center for Atmospheric Research. Typical proxy estimates for Miocene *p*CO_2_ vary between ~300 and 600 ppm (*15*). The sensitivity of our model to CO_2_ concentration was tested by forcing with *p*CO_2_ of 560 and 280 ppm. The Miocene setting includes topography and vegetation from the MioMIP2-phase 1 experimental design (*64*), while solar constant, orbital configuration, and other greenhouse gas concentrations are maintained at modern values. The simulation integrates 1400 years to reach a quasi-equilibrium state. Climate states analyzed here refer to the climatological average over the final 100 model simulation years.

## Supplementary Materials

### This PDF file includes:

Supplementary Text Figs. S1 to S7 Tables S1 and S2 References
