# Supplementary material for: Evolution of Southern Hemisphere Westerly asymmetry since the Early Miocene
Source: Sci Adv. 2026 May 27;12(22):eaee0530. doi: 10.1126/sciadv.aee0530 (PMC13215180; doi:10.1126/sciadv.aee0530)
Supplement: Supplementary file 1 — Supplementary Text Figs. S1 to S7 Tables S1 and S2 References [file sciadv.aee0530_sm.pdf]

Science Advances
AAAS

Supplementary Materials for

Evolution of Southern Hemisphere Westerly asymmetry since the Early Miocene

Congcong Gai *et al.*

Corresponding author: Huaichun Wu, whcgeo@cugb.edu.cn; Qingsong Liu, qsliu@sustech.edu.cn

Sci. Adv. **12**, eaee0530 (2026)
DOI: 10.1126/sciadv.aee0530

This PDF file includes:

Supplementary Text
Figs. S1 to S7
Tables S1 and S2
References

Supplementary Text

1. Sedimentary grain size analyses

The grain-size distribution of Site U1370 samples can be classified into 3 groups (fig. S1A): Group 1 (most of the samples) and Group 2 have unimodal grain-size distributions with peaks at ~4 μm and ~16 μm, respectively. Group 3 has a bimodal grain-size distribution with two peaks between 4 and 16 μm. Grain-size principal component analysis indicates that two endmembers (EMs) can explain 96.8% of the variance in the data set (fig. S2). EM1 (mean size = 3.47 μm) represents fine particles and is interpreted as eolian dust. Most of EM2 (mean size = 16.00 μm) is silt coarser than 10 μm. These particles are noncohesive and respond to hydrodynamic forces at the ocean bottom (*20*), which indicates that EM2 is predominantly controllled by water circulation strength. Although Antarctic-derived turbidites generally consist of fine particles (*78*), the Southeast Pacific Rise is a natural barrier to Antarctic-derived turbidites. Considering that EM2 contributes little (average ~14%) to the <5 μm size fraction (fig. S1B), the <5 μm terrestrial fraction extracted here can be used to represent the dust fraction.

Coarse fraction particles (>240 μm) consist of up to 4% abundance in a few samples (fig. S1C). The coarser fraction in pelagic sediments is usually associated with volcanic, biogenic, and/or ice-rafted materials (*79–81*). Site U1370 sediments are dominated by unfossiliferous red clay (*82*). Carbonate has been removed before analysis (see Methods), so biogenic opal could explain the presence of coarse particles. However, siliceous microfossils, including diatoms, radiolarians, and siliceous sponge spicules, are usually >10 μm in size (*83–86*), so their presence would have little effect on the <5 μm fraction extracted here. Volcanic materials usually have positive ε_{Nd} values (*26*, *87*), while elevated coarse particle abundances do not correspond systematically to shifts to higher ε_{Nd} values. Thus, the coarse particles are likely to be ice-rafted detritus (IRD). IRD is usually unsorted, as icebergs carry sediments with a wide grain size range (*88*, *89*). However, grain sizes of samples with an elevated >240 μm fraction have a well-sorted distribution, similar to the grain-size distribution of other samples (fig. S1), which suggests a negligible effect of IRD on the <5 μm fraction. To minimize the potential IRD influence, only samples with coarser fraction <1% were considered for Sr-Nd isotope analysis. Therefore, the <5 μm fraction of our samples reflects primarily a dust signal.

2. Determination of dust source endmembers

2.1 Exclusion of contributions from Antarctica, New Zealand, and Southern Africa

Terrigenous material from all Southern Hemisphere continents can be transported as eolian dust toward the South Pacific Ocean (*26*, *36*, *90*). Antarctic ice sheets have developed to near-modern volume since the early to middle Miocene (*91*) and dynamic glaciers and ice sheets efficiently produce glaciogenic fine particles delivered to the Southern Ocean (*63*). However, reconstruction of glacial and interglacial dust fluxes in the subarctic South Pacific and at Taylor Glacier near the main Antarctic dust sources indicate that Antarctica acts primarily as a dust sink (*36*, *92*, *93*). Therefore, we conclude that Antarctica provided little terrigenous material to our study area and that the <5 μm fraction at Site U1370 is dominated by eolian input from lower latitude source regions.

Site U1370 radiogenic isotope data from the <5 μm fraction were then compared with data from Australia, New Zealand, South America, and southern Africa. Site U1370 Sr-Nd isotope data are broadly linearly distributed (Fig. 2I), which can result from binary mixing of endmembers with different radiogenic Nd isotope compositions. Among the potential source areas, New Zealand is limited in extent, and is a small dust source to the South Pacific Ocean today (*94*). Past New Zealand dust emissions mainly occur during glacial periods, typically linked to lowered sea level, expanded shelves and large-scale glacial coverage on the South Island (*26*). Long-term cooling in the South Island did not start until ~3 Ma (*95*), so warm climate conditions and relatively high sea levels suggest that New Zealand was not a major dust source during the Miocene-Pliocene. Southern African dust that enters the ocean is predominantly deposited in the South Atlantic Ocean via southeast trade-winds and Berg winds that contribute little to South Pacific dust deposition (*90*, *96*, *97*). This is consistent with Sr-Nd isotope compositions of the Site U1370 dust fraction that are offset systematically from New Zealand compositions and clearly different to mixing scenarios involving southern African dust (Fig. 2I). Therefore, we exclude New Zealand and southern Africa as important dust sources to Site U1370 across the studied time interval.

2.2 Determination of East-Central Australian and Central South American endmembers

Geochemical characteristics of East-Central Australian and Central South American endmembers used in this study are shown in Fig. 2I and Table S2. Sr and Nd isotope data for the East-Central Australian endmember are from <2 μm samples in the Darling Basin (*24*). There is a lack of Sr and Nd concentration data from the Darling Basin, and data from the Tasman Sea core

E26.1 (40.28°S, 168.33°E) <10 μm fraction are used here because the eastern Tasman Sea is fed by Australian dust and has similar Sr and Nd isotopic compositions (*25*, *35*). For the Central South American endmember Nd isotope, all <5 μm samples from subregions (including the Southern Altiplano, Northern Puna, Southern Puna, North-Central Western Argentina, and Middle-Central Western Argentina) located between 19 and 32°S in (*37*) are used.

Some of these Central South American subregions only contain one or two <5 μm samples, which may lead to overrepresentations of specific subregions in the Nd isotope average. Given that Nd isotope compositions are relatively insensitive to grain size variations, up to two <63 μm samples were added to reach a minimum of three samples from each subregion to yield a robust Nd isotopic average. Nd concentration endmember constraints are based on all samples located between 19 and 32°S in (*37*). The Sr isotope composition can be affected significantly by grain size variations, which implies that sorting processes during transport may lead to higher $^{87}Sr/^{86}Sr$ values (*98*). Samples 8 and 6 with relatively high isotopic compositions in (*37*) are used to represent the Sr isotopic composition and concentration endmember, respectively.

Apart from the endmember presented above, we also tested a hypothetical scenario depicting more "extreme" case, that is, using the highest published $^{87}Sr/^{86}Sr$ and lowest ε_{Nd} values from Central South American source region (*37*) to represent an "extreme" Central South American endmember (Fig. 2I). In this scenario, Central South American dust contributed ~50% before 8.4 Ma while East-Central Australian dust contributed more than 80% since 8.4 Ma. Although the relative contribution of dust sources changed more or less, this "extreme" case also indicates a primary dust source shift from Central South America to Australia at 8.4 Ma, which indicates that our conclusion about the dust source shift is not entirely dependent on endmember composition choice.

3. Sedimentary rock magnetic characteristics of Site U1370

Magnetic minerals have been used widely to trace eolian dust in the Pacific Ocean (*82*, *99*, *100*) because they usually occur as fine particles that can achieve long atmospheric residence times and long distance transport (*21*). Detrital and biogenic magnetite (i.e., magnetofossils) co-exist in South Pacific sediment (*101*); the latter consists of single domain particles with mean-coercivity of ~40 mT (*101*, *102*). To minimize magnetofossil influences on our provenance discussion, we only focus on magnetic parameters that are insensitive to these single domain particles.

By applying a Co-based age model to Site U1370 (*66*), a distinct magnetic parameter shift is evident between ~14 and 8.4 Ma, which change little in other time intervals (Figs. 2D-2F, fig. S3). Concentration-dependent magnetic parameters, including the S-ratio ($S_{-0.1T}$), hard isothermal remanent magnetization ($HIRM_{-0.1T}$) flux, and saturation isothermal remanent magnetization (SIRM) flux, have a marked peak between ~14 and 10 Ma, which is followed by a secondary peak between 10 and 8.4 Ma (Fig. 2F, figs. S3B and S3C). At the same time, magnetic parameters sensitive to grain-size (χ_{fd}% and ARM/SIRM) have their lowest values (Fig. 2D and fig. S3A), which indicates magnetic particle coarsening. χ-T heating curves have two peaks at ~280 and 450°C, and decrease to near-zero values at ~585 °C (Fig. 2H), which are likely caused by inversion of fine-grained maghemitized magnetite to hematite and to the presence of magnetite, respectively (*103*, *104*). Positive shape factor values indicate that hysteresis loops are wasp-waisted with at least two coexisting magnetic components with strongly contrasting B_c (*69*, *105*) (Fig. 2E and fig. S4). The two components are interpreted as low-coercivity magnetite and medium-coercivity maghemitized magnetite based on χ-T characteristics. Co-occurring magnetite and maghemitized magnetite also explain higher $HIRM_{-0.1T}$ and SIRM fluxes before 8.4 Ma (figs. S3B and S3C). For samples deposited since 8.4 Ma, χ-T heating curves decrease sharply to near-zero values at ~585°C (Fig. 2G), which indicates that low-coercivity magnetite is the dominant magnetic mineral (*104*). Moreover, the mean magnetic mineral concentration is lower and magnetic grain size fluctuates more strongly after 8.4 Ma compared to before ~14 Ma (Figs. 2D and 2F, fig. S3).

4. Significance of the Ti/Al ratio

Apart from source rock characteristics, diagenesis, authigenesis, and sorting can also affect sediment elemental compositions. Given that Ti/Al is rarely affected by diagenetic alteration (*106*), the other two processes are assessed here. Authigenic clay formation can change sedimentary Al concentrations. Authigenic clay formation via reverse weathering reactions requires reactive Al and Si sources, and cations (e.g., Ca^{2+}, Mg^{2+}, K^{+}) (*107*). For IODP Expedition 329 sediments, Mg-enriched altered volcanic ash and biogenic Si are key reactants for forming authigenic clay (*108*). Modeled mass fractions of Mg-enriched volcanic ash and biogenic Si for Site U1370 are decoupled over the Cenozoic (fig. S3 in (*108*)), so we suggest that the availability of reactive Al or both reactive Al and Si limits reverse weathering. Moreover, Q-mode Factor Analysis results indicate that Al and Ti are least affected by authigenic processes (*19*). Therefore, Ti/Al at Site U1370 is considered largely unaffected by authigenic clay formation via reverse weathering. In terms of

sorting, mineralogical differentiation occurs during long-distance dust transport via gravitational sorting (*109*), and settling through the water column (*110*). Bi-plots for Site U1370 do not reveal obvious correlations between elements (Ti, Al, and Ti/Al), mean grain size or different size fractions (fig. S5), which indicates that Ti and Al are relatively evenly distributed in the different size fractions and that Ti/Al variations are unlikely to have been caused by long-range dust transport or sorting. Thus, Ti/Al here is taken to be mainly affected by source rock characteristics.

5. Control of plate tectonic movements on the dust provenance

Plate tectonics changed the position of Site U1370 relative to the main Southern Hemisphere dust sources over the last 17 Ma. The Indo-Australian Plate has shifted northward slowly while South America remained at its present location (*111*) (Fig. 1). Therefore, the relative position of Australia and Site U1370 was relatively constant while the circumpolar transport distance from Central South America to Site U1370 gradually shortened (Fig. 1 and fig. S7). If tectonic changes controlled the dust provenance, we would expect a gradual increase in the Central South American contribution at Site U1370 since the late Miocene. This contrasts with our geochemical and magnetic results.

6. Reconciliation between core MV0502-01JC and Site U1370 Sr-Nd data

Core MV0502-01JC sediment was recovered at Marlin Rise (40°S) (Fig. 1) (*44*), which is near our study area and was deposited over the past 17.5 Myr. Sr-Nd isotopic data for core MV0502-01JC detrital mineral extracts are close to our calculated mixing line (Fig. 2I) but are shifted to lower $^{87}Sr/^{86}Sr$ values. The Sr isotope composition is known to have a grain size dependency, with higher isotopic ratios in the finer fraction (*98*). Core MV0502-01JC data were measured for the <38 μm fraction, and the grain-size distribution for MV0502-01JC samples suggest that >5 μm particles are also present (*44*). We speculate that the clay mineral content in core MV0502-01JC samples is lower than that in our <5 μm samples, which may partly explain the overall less radiogenic Sr isotopic compositions in core MV0502-01JC. Also, possible incomplete removal of authigenic Nd and Sr carrying phases during leaching could have biased both the Nd and Sr isotope composition (*112*). Moreover, the age model of core MV0502-01JC was reconstructed by comparing the $^{87}Sr/^{86}Sr$ ratio of fish teeth with the seawater Sr isotope curve (*44*). Because fossil fish teeth continue to recrystallize and exchange Sr with pore water during burial and diagenesis (*113*, *114*), this dating method can yield age offsets of several million years,

which further complicates comparison between Site U1370 and core MV0502-01JC datasets through time.

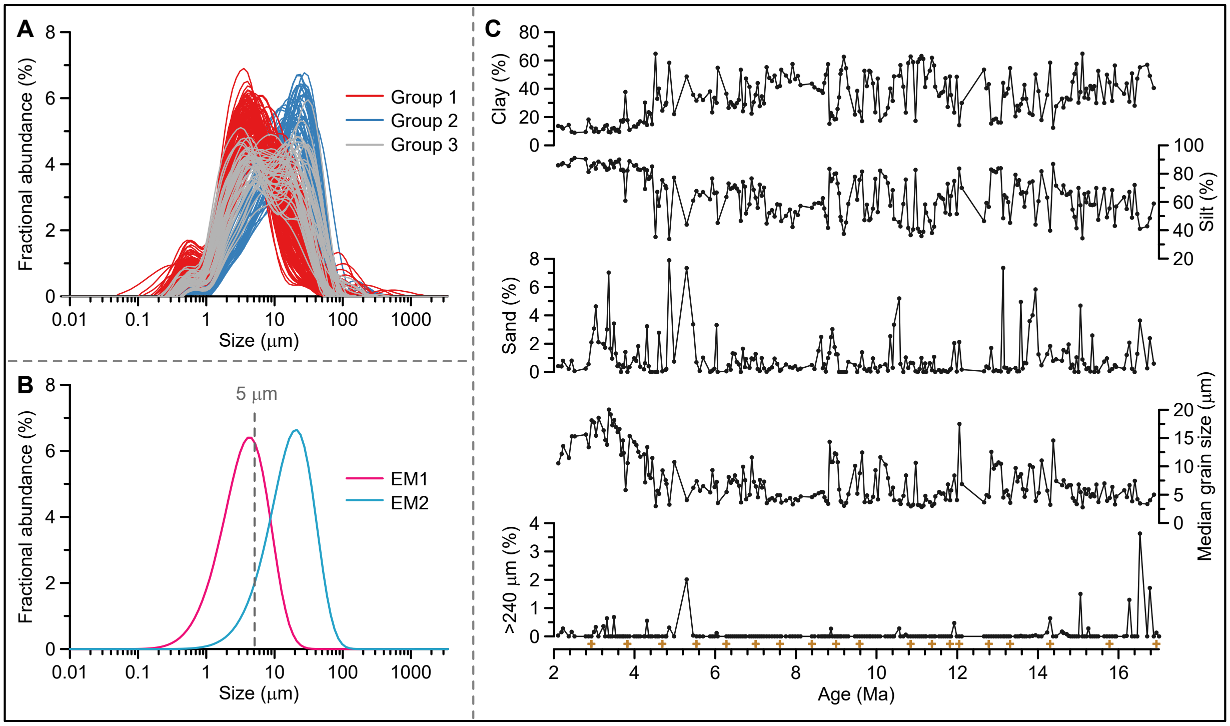

Fig. S1. Sedimentary grain size distributions and endmember analysis results from Site U1370. (**A**) Grain size distributions. (**B**) Endmembers (EMs) 1 and 2 derived from the grain-size principal component analysis. (**C**) Fractional abundances of different grain size components and median grain size. Brown crosses in (**C**) denote samples used for Sr-Nd isotope measurements.

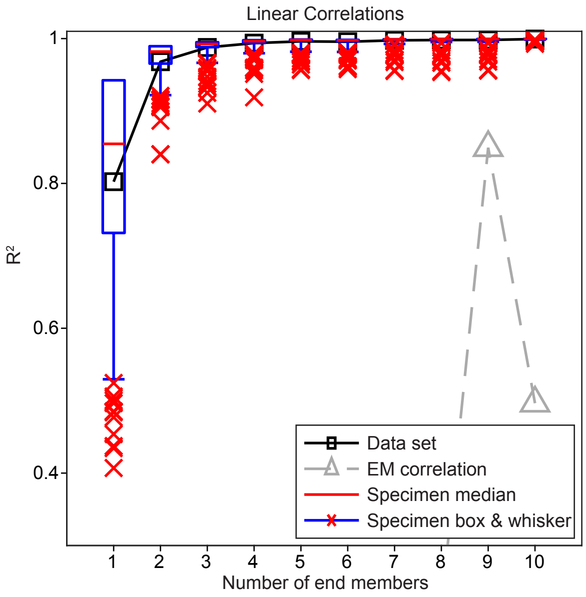

Fig. S2. Squared linear correlations between the measured data set and the fitted endmember reconstruction.

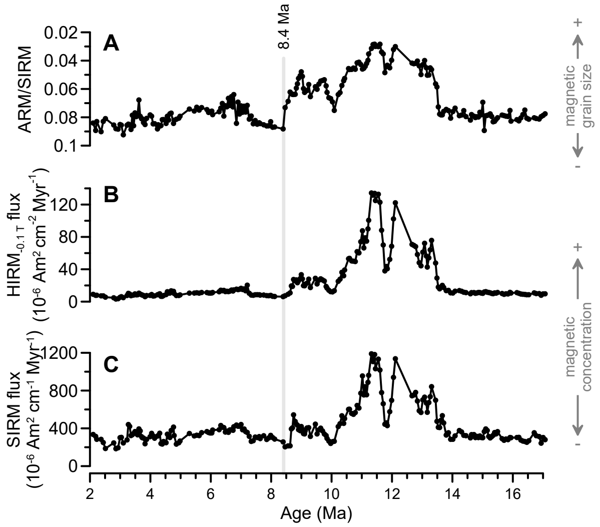

Fig. S3. Rock magnetic results from Site U1370. (**A**) ARM/SIRM. (**B**) $HIRM_{-0.1\ T}$ flux. (**C**) SIRM flux. The grey line in (**A-C**) denotes 8.4 Ma.

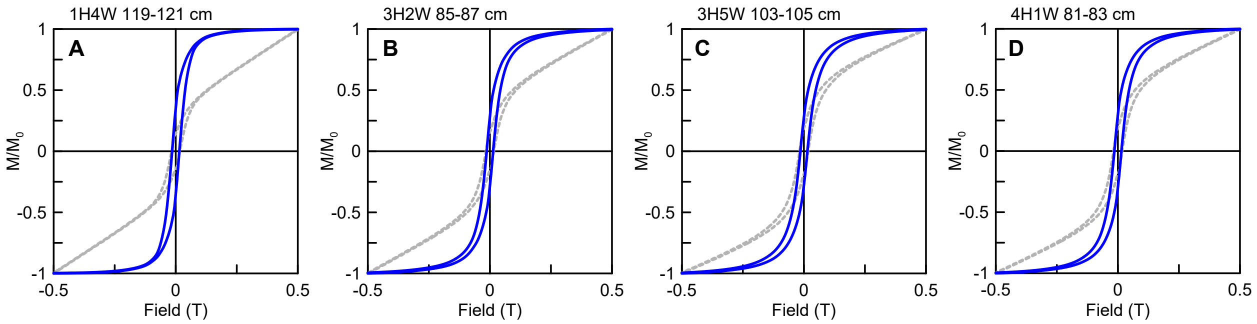

Fig. S4. Hysteresis loops of typical samples. (**A**) Pot-bellied hysteresis loops. (**B-D**) Wasp-waisted hysteresis loops.

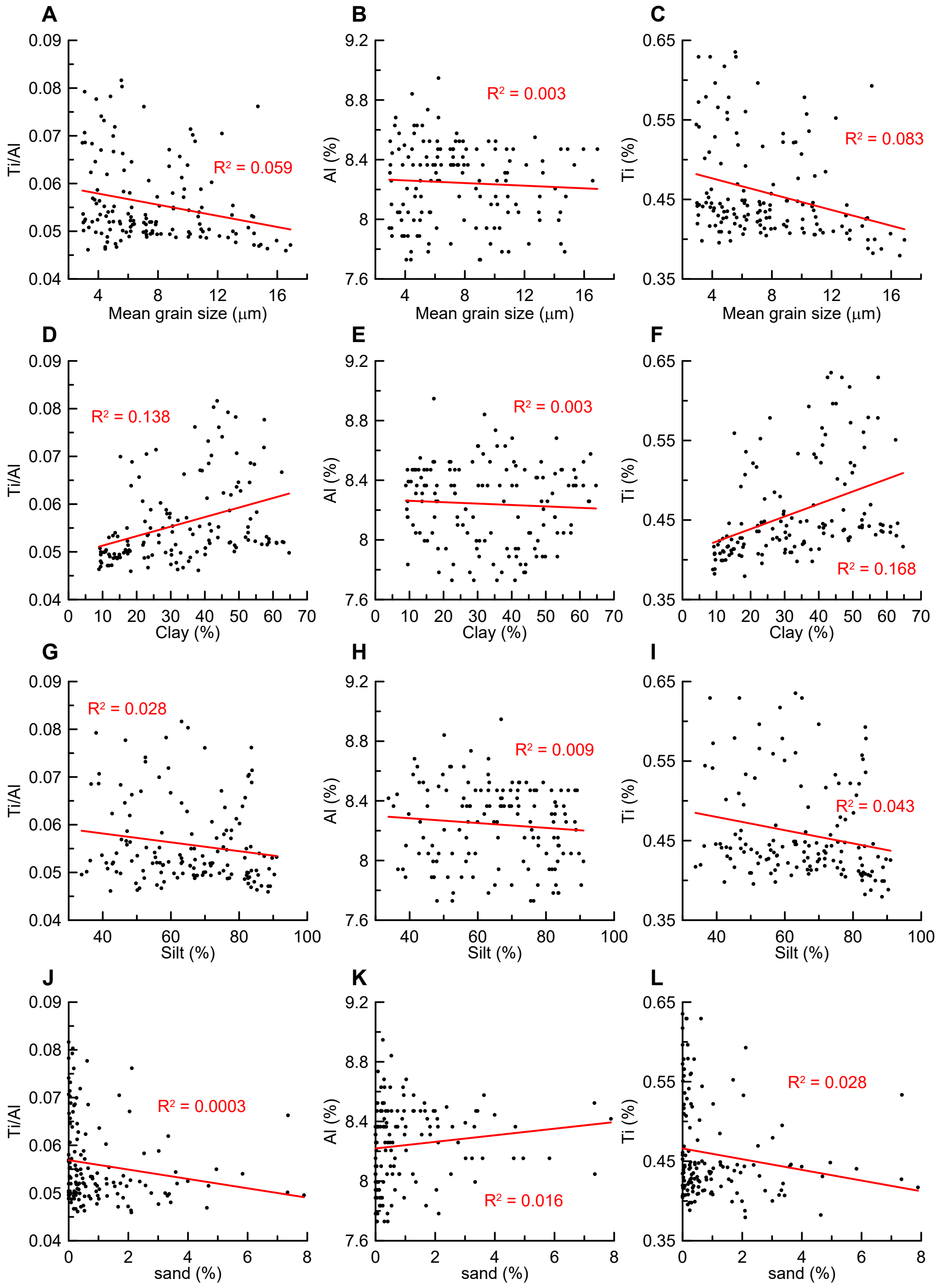

Fig. S5. Biplots of elemental and grain size parameters. (**A-C**) Biplots of mean grain size and Ti/Al, Al, and Ti, respectively. (**D-F**) Biplots of clay proportion and Ti/Al, Al, and Ti, respectively. (**G-I**) Biplots of silt proportion and Ti/Al, Al, and Ti, respectively. (**J-L**) Biplots of silt proportion and Ti/Al, Al, and Ti, respectively. Red lines are linear regression trends.

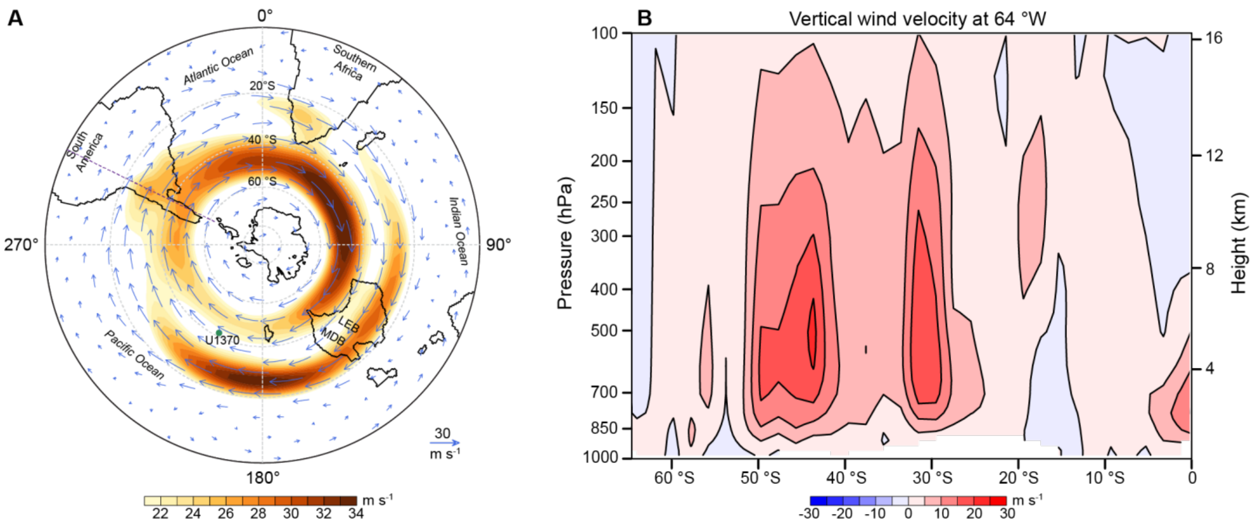

Fig. S6. Simulated Miocene 100-year annual mean of Southern Hemisphere atmospheric circulation (with 280 ppm pCO_2). (**A**) Wind vector at 200 hPa. The SHW core splits into subtropical and subpolar jets over the South Pacific Ocean. Shading indicates locations with maximum wind speeds. Blue arrows indicate the wind vector (unit: m s^{-1}); the green dot indicates the present-day location of Site U1370; the purple dashed line indicates the location of the cross-section shown in (**B**). (**B**) Latitude-pressure cross-section of vertical wind velocity (filled colors, unit: m s^{-1}) at 64 °W. Positive (negative) values represent upward (downward) velocity.

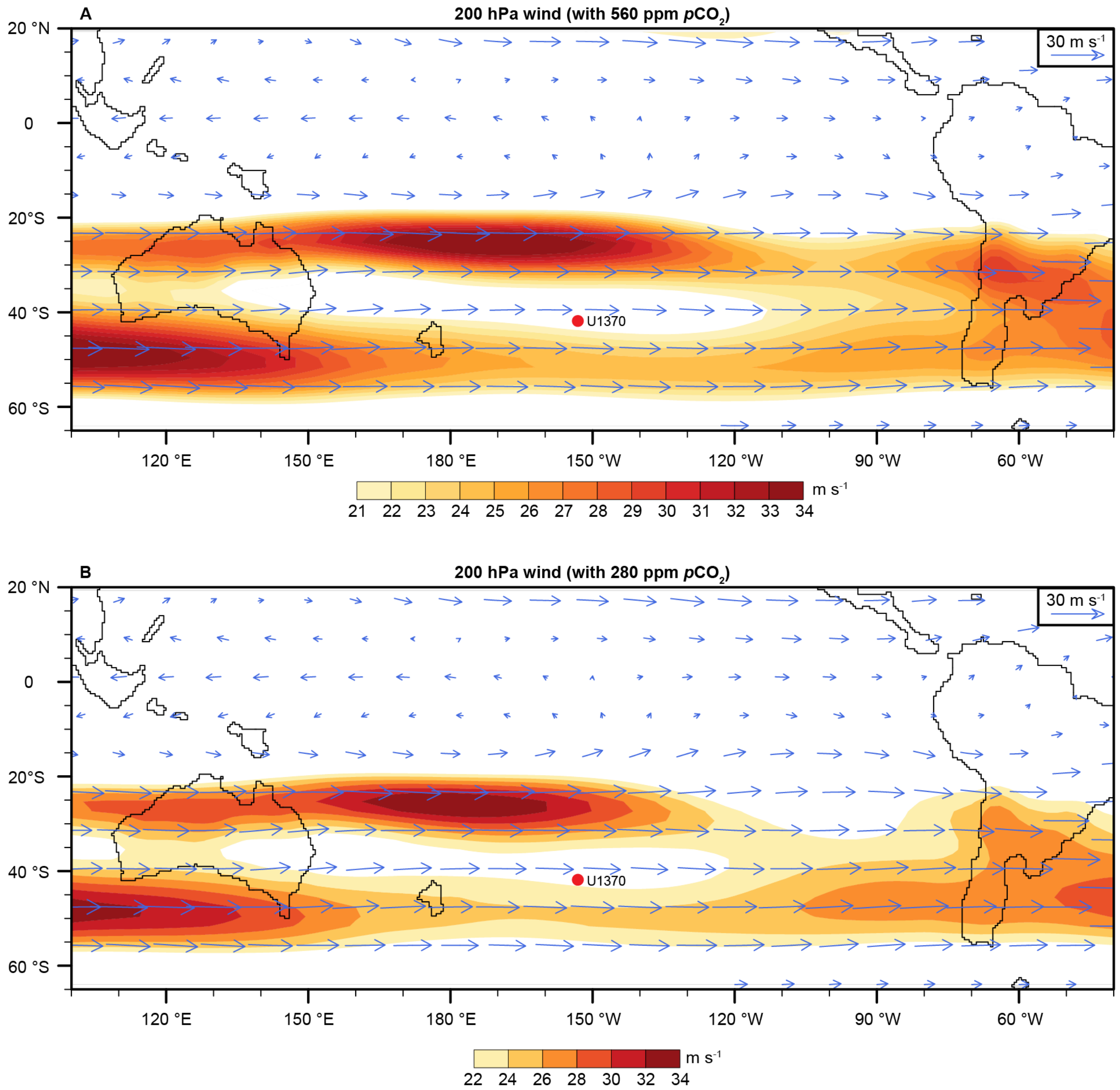

Fig. S7. Simulated middle Miocene 100-year annual mean wind vector at 200 hPa (blue arrows, unit: m s^{-1}) with (**A**) 560 ppm $p\text{CO}_2$, and (**B**) 280 ppm $p\text{CO}_2$. The red dot indicates the present-day location of Site U1370.

	ε_{Nd}	2SD	n	$^{87}Sr/^{86}Sr$	2SD	n	References
Murray Basin	-9.1	4.0	24	0.7300	0.0334	24	(*23*–*25*)
Darling Basin	-1.6	3.0	11	0.7113	0.0032	11	(*24*)
Lake Eyre Basin	-3.9	1.8	24	0.7110	0.0030	24	(*23*, *25*, *26*)
Central South America	-6.6	5.7	9	0.7157	0.0101	9	(*37*)
Southern South America	-1.0	6.9	25	0.7083	0.0051	25	(*31*, *115*)
New Zealand	-4.4	5.8	21	0.7121	0.0052	21	(*26*)
Southern Africa	-12.5	10.8	6	0.7260	0.0191	6	(*31*)

Table S1. Sr-Nd isotopic characteristics for potential Southern Hemisphere dust source areas. Values were calculated using the <5 μm fraction where available. Due to a lack of samples in the <5 μm size fraction, certain samples from Australia in (*23*, *24*) are from the <2 μm fraction.

	εNd	2SD	Nd (μg/g)	$^{87}Sr/^{86}Sr$	2SD	Sr (μg/g)	References
East-Central Australia	-1.6	3.0	25.7	0.7113	0.0032	157.0	(*24*, *25*)
Central South America	-7.4	5.8	29.4	0.7249	0.00001	156.0	(*37*)
Central South America*	-11.5	2.0	29.4	0.7316	0.00001	156.0	(*37*)
Murray Basin	-9.1	4.0	28.2	0.7300	0.0334	90	(*23–25*, *116*)

Table S2. Source data used in the isotope mixing calculation illustrated in Fig. 2.

REFERENCES

1. J. Shulmeister, I. Goodwin, J. Renwick, K. Harle, L. Armand, M. S. McGlone, E. Cook, J. Dodson, P. P. Hesse, P. Mayewski, M. Curran, The Southern hemisphere westerlies in the Australasian sector over the last glacial cycle: A synthesis. *Quat. Int.* **118-119**, 23–53 (2004).

2. A. D. Sproson, Y. Yokoyama, Y. Miyairi, T. Aze, V. J. Clementi, H. Riechelson, S. C. Bova, Y. Rosenthal, L. B. Childress, I. W. Aiello, A. Avila, W. Biggs, C. D. Charles, A. H. Cheung, K. DeLong, I. A. Dove, X. Du, E. R. Estes, U. Fuentes, C. García-Lasanta, S. L. Goldstein, A. Golub, J. R. Hagemann, R. G. Hatfield, L. L. Haynes, A. V. Hess, N. Irvali, Y. Kiro, M. M. Monteagudo, J. E. Lambert, C. Li, W. M. Longo, S. McGrath, R. S. Robinson, J. Sarao, S. Taylor, J. D. Wright, S. M. Yu, Expedition 379T Scientists, Near-synchronous Northern Hemisphere and Patagonian ice sheet variation over the last glacial cycle. *Nat. Geosci.* **17**, 450–457 (2024).

3. L. Menviel, P. Spence, J. Yu, M. A. Chamberlain, R. J. Matear, K. J. Meissner, M. H. England, Southern Hemisphere westerlies as a driver of the early deglacial atmospheric CO_2 rise. *Nat. Commun.* **9**, 2503 (2018).

4. K. A. Wendt, C. Nehrbass-Ahles, K. Niezgoda, D. Noone, M. Kalk, L. Menviel, J. Gottschalk, J. W. B. Rae, J. Schmitt, H. Fischer, T. F. Stocker, J. Muglia, D. Ferreira, S. A. Marcott, E. Brook, C. Buizert, Southern Ocean drives multidecadal atmospheric CO_2 rise during Heinrich stadials. *Proc. Natl. Acad. Sci. U.S.A.* **121**, e2319652121 (2024).

5. J. Weis, Z. Chase, C. Schallenberg, P. G. Strutton, A. R. Bowie, S. L. Fiddes, One-third of Southern Ocean productivity is supported by dust deposition. *Nature* **629**, 603–608 (2024).

6. A. Martínez-Garcia, A. Rosell-Melé, S. L. Jaccard, W. Geibert, D. M. Sigman, G. H. Haug, Southern Ocean dust-climate coupling over the past four million years. *Nature* **476**, 312–315 (2011).

7. C. Le Quéré, C. Rödenbeck, E. T. Buitenhuis, T. J. Conway, R. Langenfelds, A. Gomez, C. Labuschagne, M. Ramonet, T. Nakazawa, N. Metzl, N. Gillett, M. Heimann, Saturation of the Southern Ocean CO_2 sink due to recent climate change. *Science* **316**, 1735–1738 (2007).

8. A. Meucci, I. R. Young, M. Hemer, C. Trenham, I. G. Watterson, 140 years of global ocean wind-wave climate derived from CMIP6 ACCESS-CM2 and EC-Earth3 GCMs: Global trends, regional changes, and future projections. *J. Clim.* **36**, 1605–1631 (2023).

9. M. S. Fletcher, P. I. Moreno, Zonally symmetric changes in the strength and position of the Southern Westerlies drove atmospheric CO_2 variations over the past 14 k.y. *Geology* **39**, 419–422 (2011).

10. D. W. Waugh, A. Banerjee, J. C. Fyfe, L. M. Polvani, Contrasting recent trends in southern hemisphere westerlies across different ocean basins. *Geophys. Res. Lett.* **47**, e2020GL088890 (2020).

11. T. M. Bals-Elsholz, E. H. Atallah, L. F. Bosart, T. A. Wasula, M. J. Cempa, A. R. Lupo, The wintertime southern hemisphere split jet: Structure, variability, and evolution. *J. Clim.* **14**, 4191–4215 (2001).

12. V. Montade, M. Kageyama, N. Combourieu-Nebout, M. P. Ledru, E. Michel, G. Siani, C. Kissel, Teleconnection between the intertropical convergence zone and southern westerly winds throughout the last deglaciation. *Geology* **43**, 735–738 (2015).

13. F. Lamy, J. C. H. Chiang, G. Martínez-Méndez, M. Thierens, H. W. Arz, J. Bosmans, D. Hebbeln, F. Lambert, L. Lembke-Jene, J. B. Stuut, Precession modulation of the South Pacific westerly wind belt over the past million years. *Proc. Natl. Acad. Sci. U.S.A.* **116**, 23455–23460 (2019).

14. A. E. Shevenell, J. P. Kennett, D. W. Lea, Middle Miocene ice sheet dynamics, deep-sea temperatures, and carbon cycling: A Southern Ocean perspective. *Geochem. Geophys. Geosyst.* **9**, Q02006 (2008).

15. M. Steinthorsdottir, H. K. Coxall, A. M. de Boer, M. Huber, N. Barbolini, C. D. Bradshaw, N. J. Burls, S. J. Feakins, E. Gasson, J. Henderiks, A. E. Holbourn, S. Kiel, M. J. Kohn, G. Knorr, W. M. Kürschner, C. H. Lear, D. Liebrand, D. J. Lunt, T. Mörs, P. N. Pearson, M. J. Pound, H. Stoll, C. A. E. Strömberg, The Miocene: The future of the past. *Paleoceanogr. Paleoclimatol.* **36**, e2020PA004037 (2021).

16. J. Groeneveld, J. Henderiks, W. Renema, C. M. McHugh, D. De Vleeschouwer, B. A. Christensen, C. S. Fulthorpe, L. Reuning, S. J. Gallagher, K. Bogus, G. Auer, T. Ishiwa, Australian shelf sediments reveal shifts in Miocene Southern Hemisphere westerlies. *Sci. Adv.* **3**, e1602567 (2017).

17. F. Lamy, R. Kilian, H. W. Arz, J. P. Francois, J. Kaiser, M. Prange, T. Steinke, Holocene changes in the position and intensity of the southern westerly wind belt. *Nat. Geosci.* **3**, 695–699 (2010).

18. P. I. Moreno, W. I. Henríquez, O. H. Pesce, C. A. Henríquez, M. S. Fletcher, R. D. Garreaud, R. P. Villa-Martínez, An Early Holocene westerly minimum in the southern mid-latitudes. *Quat. Sci. Rev.* **251**, 106730 (2021).

19. A. G. Dunlea, R. W. Murray, J. Sauvage, A. J. Spivack, R. N. Harris, S. D'Hondt, Dust, volcanic ash, and the evolution of the South Pacific Gyre through the Cenozoic. *Paleoceanography* **30**, 1078–1099 (2015).

20. I. N. McCave, B. Manighetti, S. G. Robinson, Sortable silt and fine sediment size/composition slicing: Parameters for palaeocurrent speed and palaeoceanography. *Paleoceanography* **10**, 593–610 (1995).

21. B. A. Maher, The magnetic properties of Quaternary aeolian dusts and sediments, and their palaeoclimatic significance. *Aeolian Res.* **3**, 87–144 (2011).

22. P. De Deckker, An evaluation of Australia as a major source of dust. *Earth Sci. Rev.* **194**, 536–567 (2019).

23. P. De Deckker, Airborne dust traffic from Australia in modern and Late Quaternary times. *Glob. Planet. Change* **184**, 103056 (2020).

24. F. X. Gingele, P. De Deckker, Clay mineral, geochemical and Sr-Nd isotopic fingerprinting of sediments in the Murray-Darling fluvial system, southeast Australia. *Aust. J. Earth Sci.* **52**, 965–974 (2005).

25. M. Revel-Rolland, P. De Deckker, B. Delmonte, P. P. Hesse, J. W. Magee, I. Basile-Doelsch, F. Grousset, D. Bosch, Eastern Australia: A possible source of dust in East Antarctica interglacial ice. *Earth Planet. Sci. Lett.* **249**, 1–13 (2006).

26. B. G. Koffman, S. L. Goldstein, G. Winckler, A. Borunda, M. R. Kaplan, L. Bolge, Y. Cai, C. Recasens, T. N. B. Koffman, P. Vallelonga, New Zealand as a source of mineral dust to the atmosphere and ocean. *Quat. Sci. Rev.* **251**, 106659 (2021).

27. S. E. Tate, R. S. B. Greene, K. M. Scott, K. G. McQueen, Recognition and characterisation of the aeolian component in soils in the Girilambone Region, north western New South Wales, Australia. *Catena* **69**, 122–133 (2007).

28. P. P. Hesse, Mineral magnetic "tracing" of aeolian dust in southwest Pacific sediments. *Palaeogeogr. Palaeoclimatol. Palaeoecol.* **131**, 327–353 (1997).

29. R. L. Reynolds, S. R. Cattle, B. M. Moskowitz, H. L. Goldstein, K. Yauk, C. B. Flagg, T. S. Berquó, R. F. Kokaly, S. Morman, G. N. Breit, Iron oxide minerals in dust of the Red Dawn event in eastern Australia, September 2009. *Aeolian Res.* **15**, 1–13 (2014).

30. C. Hird, M. M. G. Perron, T. M. Holmes, S. Meyerink, C. Nielsen, A. T. Townsend, P. de Caritat, M. Strzelec, A. R. Bowie, On the use of lithogenic tracer measurements in aerosols to constrain dust deposition fluxes to the ocean southeast of Australia. *Aerosol Res.* **2**, 315–327 (2024).

31. B. Delmonte, I. Basile-Doelsch, J. R. Petit, V. Maggi, M. Revel-Rolland, A. Michard, E. Jagoutz, F. Grousset, Comparing the Epica and Vostok dust records during the last 220,000 years: Stratigraphical correlation and provenance in glacial periods. *Earth Sci. Rev.* **66**, 63–87 (2004).

32. A. E. Barkley, G. Winckler, C. Recasens, M. R. Kaplan, B. G. Koffman, F. Calabozo, J. L. Middleton, R. F. Anderson, Y. Cai, L. Bolge, J. Longman, S. L. Goldstein, Patagonian dust, Agulhas current, and Antarctic ice-rafted debris contributions to the South Atlantic Ocean over the past 150,000 years. *Proc. Natl. Acad. Sci. U.S.A.* **121**, e2402120121 (2024).

33. J. P. Le Roux, A review of tertiary climate changes in southern South America and the Antarctic Peninsula. Part 2: Continental conditions. *Sediment. Geol.* **247-248**, 21–38 (2012).

34. E. C. Christeleit, M. T. Brandon, D. L. Shuster, Miocene development of alpine glacial relief in the Patagonian Andes, as revealed by low-temperature thermochronometry. *Earth Planet. Sci. Lett.* **460**, 152–163 (2017).

35. T. Struve, K. Pahnke, F. Lamy, M. Wengler, P. Böning, G. Winckler, A circumpolar dust conveyor in the glacial Southern Ocean. *Nat. Commun.* **11**, 5655 (2020).

36. T. Struve, J. Longman, M. Zander, F. Lamy, G. Winckler, K. Pahnke, Systematic changes in circumpolar dust transport to the Subantarctic Pacific Ocean over the last two glacial cycles. *Proc. Natl. Acad. Sci. U.S.A.* **119**, e2206085119 (2022).

37. S. Gili, D. M. Gaiero, S. L. Goldstein, F. Chemale, J. Jweda, M. R. Kaplan, R. A. Becchio, E. Koester, Glacial/interglacial changes of Southern Hemisphere wind circulation from the geochemistry of South American dust. *Earth Planet. Sci. Lett.* **469**, 98–109 (2017).

38. C. A. Tapia, G. S. Wilson, Rock magnetic properties and paleomagnetic behavior of Neogene marine sediments from northern Chile. *Geochem. Geophys. Geosyst.* **15**, 4400–4423 (2014).

39. B. Carter-Stiglitz, S. K. Banerjee, A. Gourlan, E. Oches, A multi-proxy study of Argentina loess: Marine oxygen isotope stage 4 and 5 environmental record from pedogenic hematite. *Palaeogeogr. Palaeoclimatol. Palaeoecol.* **239**, 45–62 (2006).

40. P. M. Vasconcelos, M. Reich, D. L. Shuster, The paleoclimatic signatures of supergene metal deposits. *Elements* **11**, 317–322 (2015).

41. S. Muñoz-Farías, B. Ritter, T. J. Dunai, J. Morales-Leal, E. Campos, R. Spikings, R. Riquelme, Geomorphological significance of the Atacama Pediplain as a marker for the climatic and tectonic evolution of the Andean forearc, between 26° to 28°S. *Geomorphology* **420**, 108504 (2023).

42. G. M. Young, H. W. Nesbitt, Processes controlling the distribution of Ti and Al in weathering profiles, siliciclastic sediments and sedimentary rocks. *J. Sediment. Res.* **68**, 448–455 (1998).

43. D. S. Vandervoort, T. E. Jordan, P. K. Zeitler, R. N. Alonso, Chronology of internal drainage development and uplift, southern Puna plateau, Argentine central Andes. *Geology* **23**, 145–148 (1995).

44. A. M. Stancin, J. D. Gleason, S. A. Hovan, D. K. Rea, R. M. Owen, T. C. Moore, C. M. Hall, J. D. Blum, Miocene to recent eolian dust record from the Southwest Pacific Ocean at 40° S latitude. *Palaeogeogr. Palaeoclimatol. Palaeoecol.* **261**, 218–233 (2008).

45. R. N. Alonso, T. E. Jordan, K. T. Tabbutt, D. S. Vandervoort, Giant evaporite belts of the Neogene central Andes. *Geology* **19**, 401–404 (1991).

46. G. H. McTainsh, R. Burgess, J. R. Pitblado, Aridity, drought and dust storms in Australia (1960–84). *J. Arid Environ.* **16**, 11–22 (1989).

47. S. Pichat, W. Abouchami, S. J. G. Galer, Lead isotopes in the Eastern Equatorial Pacific record Quaternary migration of the South Westerlies. *Earth Planet. Sci. Lett.* **388**, 293–305 (2014).

48. J. C. H. Chiang, S. Y. Lee, A. E. Putnam, X. Wang, South Pacific split jet, ITCZ shifts, and atmospheric north-south linkages during abrupt climate changes of the last glacial period. *Earth Planet. Sci. Lett.* **406**, 233–246 (2014).

49. A. Y. Hou, R. S. Lindzen, The influence of concentrated heating on the Hadley circulation. *J. Atmos. Sci.* **49**, 1233–1241 (1992).

50. P. Wang, B. Wang, H. Cheng, J. Fasullo, Z. Guo, T. Kiefer, Z. Liu, The global monsoon across timescales: Coherent variability of regional monsoons. *Clim. Past.* **10**, 2007–2052 (2014).

51. S. Wan, A. Li, P. D. Clift, J. B. W. Stuut, Development of the East Asian monsoon: Mineralogical and sedimentologic records in the northern South China Sea since 20 Ma. *Palaeogeogr. Palaeoclimatol. Palaeoecol.* **254**, 561–582 (2007).

52. G. Wei, X. H. Li, Y. Liu, L. Shao, X. Liang, Geochemical record of chemical weathering and monsoon climate change since the Early Miocene in the South China Sea. *Paleoceanography* **21**, PA4214 (2006).

53. M. W. Lyle, P. A. Wilson, T. R. Janecek, J. Backman, W. H. Busch, H. K. Coxall, K. Faul, P. Gaillot, S. A. Hovan, P. Knoop, S. Kruse, L. Lanci, C. Lear, T. C. Moore, C. A. Nigrini, H. Nishi, R. Nomura, R. D. Norris, H. Palike, J. M. Pares, L. Quintin, I. Raffi, B. R. Rea, D. K. Rea, T. H. Steiger, A. Tripati, M. D. Van Den Berg, B. Wade, Leg 199 Summary, *Proceedings of the Ocean Drilling Program, 199 Init. Repts*. vol. 199, pp. 1–89.

54. J. Kim, K. Hyeong, H. S. Jung, J. W. Moon, K. H. Kim, I. Lee, Southward shift of the Intertropical Convergence Zone in the western Pacific during the Late Tertiary: Evidence from ferromanganese crusts on seamounts west of the Marshall Islands. *Paleoceanography* **21**, PA4218 (2006).

55. J. C. H. Chiang, C. M. Bitz, Influence of high latitude ice cover on the marine intertropical convergence zone. *Clim. Dyn.* **25**, 477–496 (2005).

56. E. J. Rohling, J. Yu, D. Heslop, G. L. Foster, B. Opdyke, A. P. Roberts, Sea level and deep-sea temperature reconstructions suggest quasi-stable states and critical transitions over the past 40 million years. *Sci. Adv.* **7**, eabf5326 (2021).

57. H. C. Larsen, A. D. Saunders, P. D. Clift, J. Beget, W. Wei, S. Spezzaferri, J. Ali, H. Cambray, A. Demant, G. Fitton, M. S. Fram, K. Fukuma, J. Gieskes, M. A. Holmes, J. Hunt, C. Lacasse, L. M. Larsen, H. Lykke-Andersen, A. Meltser, M. L. Morrison, N. Nemoto, N. Okay, S. Saito, C. Sinton, R. Stax, T. L. Vallier, D. Vandamme, R. Werner, Seven million years of glaciation in Greenland. *Science* **264**, 952–955 (1994).

58. H. H. Aumann, S. Broberg, E. M. Manning, Northward shift and narrowing of the ITCZ in 20 years of AIRS data. *J. Geophys. Res. Atmos.* **129**, e2023JD038723 (2024).

59. T. DeVries, The oceanic anthropogenic CO_2 sink: Storage, air-sea fluxes, and transports over the industrial era. *Global Biogeochem. Cycles* **28**, 631–647 (2014).

60. J. R. Toggweiler, J. L. Russell, S. R. Carson, Midlatitude westerlies, atmospheric CO_2, and climate change during the ice ages. *Paleoceanography* **21**, PA2005 (2006).

61. J. Zan, B. A. Maher, X. Fang, T. Stevens, W. Ning, F. Wu, Y. Yang, J. Kang, Z. Hu, Global dust impacts on biogeochemical cycles and climate. *Nat. Rev. Earth Environ.* **6**, 789–807 (2025).

62. E. M. Shoenfelt, J. Sun, G. Winckler, M. R. Kaplan, A. L. Borunda, K. R. Farrell, P. I. Moreno, D. M. Gaiero, C. Recasens, R. N. Sambrotto, B. C. Bostick, High particulate iron(II) content in glacially sourced dusts enhances productivity of a model diatom. *Sci. Adv.* **3**, e1700314 (2017).

63. E. M. Shoenfelt, G. Winckler, F. Lamy, R. F. Anderson, B. C. Bostick, Highly bioavailable dust-borne iron delivered to the Southern Ocean during glacial periods. *Proc. Natl. Acad. Sci. U.S.A.* **115**, 11180–11185 (2018).

64. N. J. Burls, C. D. Bradshaw, A. M. De Boer, N. Herold, M. Huber, M. Pound, Y. Donnadieu, A. Farnsworth, A. Frigola, E. Gasson, A. S. von der Heydt, D. K. Hutchinson, G. Knorr, K. T. Lawrence, C. H. Lear, X. Li, G. Lohmann, D. J. Lunt, A. Marzocchi, M. Prange, C. A. Riihimaki, A. C. Sarr, N. Siler, Z. Zhang, Simulating Miocene Warmth: Insights from an opportunistic multi-model ensemble (MioMIP1). *Paleoceanogr. Paleoclimatol.* **36**, e2020PA004054 (2021).

65. S. D'Hondt, F. Inagaki, C. A. Alvarez Zarikian, H. Evans, N. Dubois, T. Engelhardt, T. Ferdelman, B. Gribsholt, R. N. Harris, B. W. Hoppie, J.-H. Hyun, J. Kallmeyer, J. Kim, J. E. Lynch, M. Satoshi, Y. Morono, R. W. Murray, B. K. Reese, T. Shimono, F. Shiraishi, D. C. Smith, C. E. Smith-Duque, A. J. Spivack, B. O. Steinsbu, Y. Suzuki, M. Szpak, L. Toffin, G. Uramoto, Y. Yamaguchi, G. Zhang, X.-H. Zhang, W. Ziebis, Site U1370. *Proc. Int. Ocean Discov. Progr.* **329**, 10.2204/iodp.proc.329.108.2011 (2011).

66. A. G. Dunlea, R. W. Murray, J. Sauvage, R. A. Pockalny, A. J. Spivack, R. N. Harris, S. D'Hondt, Cobalt-based age models of pelagic clay in the South Pacific Gyre. *Geochem. Geophys. Geosyst.* **16**, 2694–2710 (2015).

67. S. G. Robinson, The Late Pleistocene palaeoclimatic record of North Atlantic deep-sea sediments revealed by mineral-magnetic measurements. *Phys. Earth Planet. Inter.* **42**, 22–47 (1986).

68. J. Bloemendal, J. W. King, F. R. Hall, S. J. Doh, Rock magnetism of Late Neogene and Pleistocene deep-sea sediments: Relationship to sediment source, diagenetic processes, and sediment lithology. *J. Geophys. Res.* **97**, 4361–4375 (1992).

69. K. Fabian, Some additional parameters to estimate domain state from isothermal magnetization measurements. *Earth Planet. Sci. Lett.* **213**, 337–345 (2003).

70. N. Dubois, N. C. Mitchell, I. R. Hall, Data report: Particle size distribution for IODP Expedition 329 sites in the South Pacific Gyre. *Proc. Int. Ocean Discov. Progr.* **329**, 10.2204/iodp.proc.329.201.2014 (2014).

71. G. A. Paterson, D. Heslop, New methods for unmixing sediment grain size data. *Geochem. Geophys. Geosyst.* **16**, 4494–4506 (2015).

72. M. F. Thirlwall, Long-term reproducibility of multicollector Sr and Nd isotope ratio analysis. *Chem. Geol.* **94**, 85–104 (1991).

73. A. Fourny, D. Weis, J. S. Scoates, Comprehensive Pb-Sr-Nd-Hf isotopic, trace element, and mineralogical characterization of mafic to ultramafic rock reference materials. *Geochem. Geophys. Geosyst.* **17**, 739–773 (2016).

74. T. Tanaka, S. Togashi, H. Kamioka, H. Amakawa, H. Kagami, T. Hamamoto, M. Yuhara, Y. Orihashi, S. Yoneda, H. Shimizu, T. Kunimaru, K. Takahashi, T. Yanagi, T. Nakano, H. Fujimaki, R. Shinjo, Y. Asahara, M. Tanimizu, C. Dragusanu, JNdi-1: A neodymium isotopic reference in consistency with LaJolla neodymium. *Chem. Geol.* **168**, 279–281 (2000).

75. S. B. Jacobsen, G. J. Wasserburg, Sm-Nd isotopic evolution of chondrites. *Earth Planet. Sci. Lett.* **50**, 139–155 (1980).

76. L. Li, Y. Yu, Y. Tang, P. Lin, J. Xie, M. Song, L. Dong, T. Zhou, L. Liu, L. Wang, Y. Pu, X. Chen, L. Chen, Z. Xie, H. Liu, L. Zhang, X. Huang, T. Feng, W. Zheng, K. Xia, H. Liu, J. Liu, Y. Wang, L. Wang, B. Jia, F. Xie, B. Wang, S. Zhao, Z. Yu, B. Zhao, J. Wei, The Flexible Global Ocean-Atmosphere-Land System Model Grid-Point Version 3 (FGOALS-g3): Description and evaluation. *J. Adv. Model. Earth Syst.* **125**, e2020JD032574 (2020).

77. M. Seton, R. D. Müller, S. Zahirovic, C. Gaina, T. Torsvik, G. Shephard, A. Talsma, M. Gurnis, M. Turner, S. Maus, M. Chandler, Global continental and ocean basin reconstructions since 200 Ma. *Earth Sci. Rev.* **113**, 212–270 (2012).

78. M. Rebesco, R. D. Larter, A. Camerlenghi, P. F. Barker, Giant sediment drifts on the continental rise west of the Antarctic Peninsula. *Geo-Mar. Lett.* **16**, 65–75 (1996).

79. L. Carter, H. L. Neil, L. Northcote, Late Quaternary ice-rafting events in the SW Pacific Ocean, off eastern New Zealand. *Mar. Geol.* **191**, 19–35 (2002).

80. B. W. Hayward, H. Neil, R. Carter, H. R. Grenfell, J. J. Hayward, Factors influencing the distribution patterns of recent deep-sea benthic foraminifera, east of New Zealand, Southwest Pacific Ocean. *Mar. Micropaleontol.* **46**, 139–176 (2002).

81. C. Saukel, F. Lamy, J. B. W. Stuut, R. Tiedemann, C. Vogt, Distribution and provenance of wind-blown SE Pacific surface sediments. *Mar. Geol.* **280**, 130–142 (2011).

82. T. Shimono, T. Yamazaki, Environmental rock-magnetism of Cenozoic red clay in the South Pacific Gyre. *Geochem. Geophys. Geosyst.* **17**, 1296–1311 (2016).

83. R. S. Pugh, I. N. McCave, Particle size measurement of diatoms with inference of their properties: Comparison of three techniques. *J. Sediment. Res.* **81**, 600–610 (2011).

84. M. R. Dennett, D. A. Caron, A. F. Michaels, S. M. Gallager, C. S. Davis, Video plankton recorder reveals high abundances of colonial Radiolaria in surface waters of the central North Pacific. *J. Plankton Res.* **24**, 797–805 (2002).

85. G. Croce, A. Frache, M. Milanesio, L. Marchese, M. Causà, D. Viterbo, A. Barbaglia, V. Bolis, G. Bavestrello, C. Cerrano, U. Benatti, M. Pozzolini, M. Giovine, H. Amenitsch, Structural characterization of siliceous spicules from marine sponges. *Biophys. J.* **86**, 526–534 (2004).

86. M. J. Uriz, X. Turon, M. A. Becerro, G. Agell, Siliceous spicules and skeleton frameworks in sponges: Origin, diversity, ultrastructural patterns, and biological functions. *Microsc. Res. Tech.* **62**, 279–299 (2003).

87. I. Basile, J. R. Petit, S. Touron, F. E. Grousset, N. Barkov, Volcanic layers in Antarctic (Vostok) ice cores: Source identification and atmospheric implications. *J. Geophys. Res. Atmos.* **106**, 31915–31931 (2001).

88. M. van der Does, F. Lamy, S. Krätschmer, J.-B. W. Stuut, C. Völker, M. Werner, R. Schwarz, M. Fleisher, G. Winckler, Late Holocene dust deposition fluxes over the entire South Atlantic Ocean. *Geochem. Geophys. Geosyst.* **25**, e2023GC011105 (2024).

89. R. McKay, J. Cockrell, A. E. Shevenell, J. S. Laberg, J. Burns, M. Patterson, S. Kim, T. Naish, D. Harwood, R. Levy, J. Marschalek, T. van de Flierdt, I. Saki, B. Keisling, I. M. C. de Sousa, G. Cortese, F. Sangiorgi, R. M. Leckie, J. Dodd, B. Duncan, L. F. Pérez, B. W. Romans, S. Kim, S. Bombard, I. Browne, T. van Peer, O. Seki, F. Colleoni, D. Kulhanek, L. De Santis, IODP Expedition 374 Science Team, Miocene ice sheet dynamics and sediment deposition in the central Ross Sea, Antarctica. *GSA. Bull.* **137**, 1267–1291 (2025).

90. J. Longman, T. Struve, K. Pahnke, Spatial and temporal trends in mineral dust provenance in the South Pacific—Evidence from mixing models. *Paleoceanogr. Paleoclimatol.* **37**, e2021PA004356 (2022).

91. E. J. Rohling, G. L. Foster, T. M. Gernon, K. M. Grant, D. Heslop, F. D. Hibbert, A. P. Roberts, J. Yu, Comparison and synthesis of sea-level and deep-sea temperature variations over the past 40 million years. *Rev. Geophys.* **60**, e2022RG000775 (2022).

92. S. M. Aarons, S. M. Aciego, C. A. Arendt, M. A. Blakowski, A. Steigmeyer, P. Gabrielli, M. R. Sierra-Hernández, E. Beaudon, B. Delmonte, G. Baccolo, N. W. May, K. A. Pratt, Dust composition changes from Taylor Glacier (East Antarctica) during the last glacial-interglacial transition: A multi-proxy approach. *Quat. Sci. Rev.* **162**, 60–71 (2017).

93. F. Lamy, R. Gersonde, G. Winckler, O. Esper, A. Jaeschke, G. Kuhn, J. Ullermann, A. Martinez-Garcia, F. Lambert, R. Kilian, Increased dust deposition in the Pacific Southern Ocean during glacial periods. *Science* **343**, 403–407 (2014).

94. M. Wengler, F. Lamy, T. Struve, A. Borunda, P. Böning, W. Geibert, G. Kuhn, K. Pahnke, J. Roberts, R. Tiedemann, G. Winckler, A geochemical approach to reconstruct modern dust fluxes and sources to the South Pacific. *Geochim. Cosmochim. Acta* **264**, 205–223 (2019).

95. R. M. Carter, P. Gammon, New Zealand maritime glaciation: Millennial-scale southern climate change since 3.9 Ma. *Science* **304**, 1659–1662 (2004).

96. D. R. Muhs, The geologic records of dust in the Quaternary. *Aeolian Res.* **9**, 3–48 (2013).

97. S. Gili, A. Vanderstraeten, A. Chaput, J. King, D. M. Gaiero, B. Delmonte, P. Vallelonga, P. Formenti, C. Di Biagio, M. Cazanau, E. Pangui, J.-F. Doussin, N. Mattielli, South African dust contribution to the high southern latitudes and East Antarctica during interglacial stages. *Commun. Earth Environ.* **3**, 129 (2022).

98. J. Smith, D. Vance, R. A. Kemp, C. Archer, P. Toms, M. King, M. Zárate, Isotopic constraints on the source of Argentinian loess - with implications for atmospheric circulation and the provenance of Antarctic dust during recent glacial maxima. *Earth Planet. Sci. Lett.* **212**, 181–196 (2003).

99. C. Gai, J. Wu, A. P. Roberts, D. Heslop, E. J. Rohling, Z. Shi, J. Liu, Y. Zhong, Y. Liu, Q. Liu, Heterogenous westerly shifts linked to Atlantic meridional overturning circulation slowdowns. *Commun. Earth Environ.* **4**, 325 (2023).

100. T. Yamazaki, N. Ioka, Environmental rock-magnetism of pelagic clay: Implications for Asian eolian input to the North Pacific since the Pliocene. *Paleoceanography* **12**, 111–124 (1997).

101. T. Yamazaki, T. Shimono, Abundant bacterial magnetite occurrence in oxic red clay. *Geology* **41**, 1191–1194 (2013).

102. T. Yamazaki, W. Fu, T. Shimono, Y. Usui, Unmixing biogenic and terrigenous magnetic mineral components in red clay of the Pacific Ocean using principal component analyses of first-order reversal curve diagrams and paleoenvironmental implications. *Earth Planets Space* **72**, 120 (2020).

103. Q. Liu, C. Deng, Y. Yu, J. Torrent, M. J. Jackson, S. K. Banerjee, R. Zhu, Temperature dependence of magnetic susceptibility in an argon environment: Implications for pedogenesis of Chinese loess/palaeosols. *Geophys. J. Int.* **161**, 102–112 (2005).

104. D. J. Dunlop, Ö. Özdemir, *Rock Magnetism: Fundamentals and Frontiers* (Cambridge Univ. Press, 1997).

105. A. P. Roberts, Y. Cui, K. L. Verosub, Wasp-waisted hysteresis loops: Mineral magnetic characteristics and discrimination of components in mixed magnetic systems. *J. Geophys. Res.* **100**, 17909–17924 (1995).

106. K. Yamamoto, R. Sugisaki, F. Arai, Chemical aspects of alteration of acidic tuffs and their application to siliceous deposits. *Chem. Geol.* **55**, 61–76 (1986).

107. F. T. Mackenzie, R. M. Garrels, Chemical mass balance between rivers and oceans. *Am. J. Sci.* **264**, 507–525 (1966).

108. A. G. Dunlea, R. W. Murray, D. P. Santiago Ramos, J. A. Higgins, Cenozoic global cooling and increased seawater Mg/Ca via reduced reverse weathering. *Nat. Commun.* **8**, 1–7 (2017).

109. H. Tsoar, K. Pye, Dust transport and the question of desert loess formation. *Sedimentology* **34**, 139–153 (1987).

110. I. N. McCave, I. R. Hall, Size sorting in marine muds: Processes, pitfalls, and prospects for paleoflow-speed proxies. *Geochem. Geophys. Geosyst.* **7**, Q10N05 (2006).

111. C. R. Scotese, An atlas of phanerozoic paleogeographic maps: The seas come in and the seas go out. *Annu. Rev. Earth Planet. Sci.* **49**, 679–728 (2021).

112. J. D. H. Williams, T. Mayer, J. O. Nriagu, Extractability of phosphorus from phosphate minerals common in soils and sediments. *Soil Sci. Soc. Am. J.* **44**, 462–465 (1980).

113. E. E. Martin, H. D. Scher, Preservation of seawater Sr and Nd isotopes in fossil fish teeth: Bad news and good news. *Earth Planet. Sci. Lett.* **220**, 25–39 (2004).

114. J. D. Gleason, T. C. Moore, D. K. Rea, T. M. Johnson, R. M. Owen, J. D. Blum, S. A. Hovan, C. E. Jones, Ichthyolith strontium isotope stratigraphy of a Neogene red clay sequence: Calibrating eolian dust accumulation rates in the central North Pacific. *Earth Planet. Sci. Lett.* **202**, 625–636 (2002).

115. F. E. Grousset, P. E. Biscaye, M. Revel, J. R. Petit, K. Pye, S. Joussaume, J. Jouzel, Antarctic (Dome C) ice-core dust at 18 k.y. B.P.: Isotopic constraints on origins. *Earth Planet. Sci. Lett.* **111**, 175–182 (1992).

116. S. K. Marx, B. S. Kamber, Trace-element systematics of sediments in the Murray-Darling Basin, Australia: Sediment provenance and palaeoclimate implications of fine scale chemical heterogeneity. *Appl. Geochem.* **25**, 1221–1237 (2010).
